# The Impact of Zinc on T Cell Motility and the Immunological Synapse

**DOI:** 10.3390/ijms27125249

**Published:** 2026-06-10

**Authors:** Atlantida Dermaku, Hannah Schoofs, Lothar Rink, Henrike Josephine Fischer

**Affiliations:** 1 Institute of Immunology, Medical Faculty, RWTH Aachen University, Pauwelstrasse 30, 52074 Aachen, Germany; atlantida.dermaku@rwth-aachen.de (A.D.); hschoofs@ukaachen.de (H.S.); henrike.fischer@pei.de (H.J.F.); 2 Paul-Ehrlich-Institut, Paul-Ehrlich-Str. 51–59, 63225 Langen, Germany

**Keywords:** Zinc, FAK, ERM, CD49d, T cell-motility, immunological synapse, LFA-1

## Abstract

Zinc is an essential trace element with a critical role in regulating immune functions. Patients with autoimmune diseases or chronic lymphatic leukemia often exhibit lower serum zinc levels. As T cells are key mediators of adaptive immunity, disturbances in zinc homeostasis can strongly affect their function. Effective T cell activity depends on directed migration to inflamed tissues, requiring coordinated cytoskeletal reorganization. This process involves the formation of a leading edge and a trailing edge (uropod) and is regulated by the ezrin–radixin–moesin (ERM) complex and its interaction with focal adhesion kinase (FAK). We investigated how zinc availability influences the expression and phosphorylation of FAK and ERM, as well as other migration-related molecules, including LFA-1 and the CD49d/CD44 complex, using Western blot, qRT-PCR, and flow cytometry in the HUT78 T cell line. Cells were cultured in media with different zinc concentrations. Zinc deficiency reduced FAK and ERM expression and decreased LFA-1 while increasing CD49d expression. Overall, these findings indicate that zinc deficiency compromises cytoskeletal remodeling and may impair T cell motility. Maintaining zinc homeostasis could thus enhance T cell migration and strengthen immune responsiveness, highlighting the potential therapeutic relevance of zinc in immune modulation.

## 1. Introduction

Zinc is a trace element known to play an important role in immune system function. Individuals with low serum zinc levels are more susceptible to infections and have a higher incidence of autoimmune disorders [1,2,3,4]. Previous research indicates that disrupted zinc homeostasis affects many immune functions of both innate and adaptive immunity, including impaired maturation and lymphocyte proliferation, as well as increased production of pro-inflammatory cytokines due to zinc deficiency [5,6]. Since altered zinc homeostasis, especially zinc deficiency, can impair several immunological responses, we decided to investigate the potential effect of disrupted zinc homeostasis on T cell motility, as T cell migration to inflammation sites is a key part of proper immune responses [7].

The migration of T cells is a tightly regulated, multi-step process coordinated by numerous intracellular and membrane-associated proteins. Upon activation, T cells undergo extensive cytoskeleton remodeling to establish a leading edge, also referred to as a chemopod, enriched in chemoreceptors, and a trailing edge, or uropod, which concentrates adhesion molecules, signaling receptors, and cytoskeletal components [8,9]. The rearrangement leads to increased concentration of several proteins within the uropod, including ERM [10,11,12]. The ERM proteins are one of the main mechanical linkers between the plasma membrane and the actin cytoskeleton [13]. ERM family members have also been suggested to promote T cell activation and play a role in the formation of the immunological synapse [14,15]. In addition to their structural roles, ERM proteins contribute to T cell activation and participate in the formation of the immunological synapse [16,17]. ERM proteins interact with the FERM domain of FAK, promoting its phosphorylation and activation. FAK plays a key role in several immunological processes, including T cell migration and formation of the immunological synapse [18,19]. FAK remains autoinhibited through an intramolecular interaction between its FERM and kinase domains. This inhibition is relieved by tyrosine phosphorylation, mediated in part by LFA-1, a heterodimer consisting of CD11a and CD18 [20]. Furthermore, CD29 (encoded by ITGB1), which forms the heterodimeric very late antigen 4 (VLA-4) with CD49d, also contributes to focal adhesion by inducing tyrosine phosphorylation of FAK [21]. FAK has been previously described to stabilize the cytoskeleton of T cells by interacting with ezrin and, consequently, the ERM complex through its FERM domain [22]. Another protein that interacts with FAK’s FERM domain is talin-1 (TLN-1). TLN-1 serves as a central protein of focal adhesion complexes, thus playing a key role in the activation and connection of integrins to the actin cytoskeleton. The F3 subdomain of TLN-1 binds to FAK, while the R9 and R12 subunits shield the binding sites of the FERM domain [23].

The motility of T cells is regulated in part by interactions between lymphocyte function-associated antigen 1 (LFA-1) and intercellular adhesion molecule 1 (ICAM-1), also known as CD54. ICAM-1 is expressed at low levels on leukocytes under resting conditions, with expression markedly increasing upon cellular activation. The LFA-1–ICAM-1 interaction coordinates spatially compartmentalized adhesion and de-adhesion events within migrating T cells, processes that are crucial for proper uropod function [24,25]. This interaction initiates and supports T cell motility by enabling crawling movement and enhancing migration speed [26,27]. LFA-1 is also essential for the formation of the immunological synapse and contributes specifically to the intravascular crawling movements of T cells [28,29].

Extravascular T cell migration is primarily mediated by VLA-4 through its interaction with vascular cell adhesion molecule 1 (VCAM-1). As previously discussed, VLA-4 comprises two subunits, CD49d and CD29, with CD49d playing a predominant role in this interaction [30,31,32]. This mechanistic understanding of T cell migration has been successfully translated into clinical applications, with VLA-4 antagonists used to inhibit leukocyte trafficking. Notably, the CD49d-targeting antibody natalizumab represents a key therapeutic agent in the treatment of multiple sclerosis [32,33,34]. CD49d can associate with CD44 to form a signaling complex that modulates cytoskeletal rearrangement via regulation of ezrin and FAK. Within this complex, CD44 interacts with FAK, whereas CD49d engages with ezrin, linking extracellular adhesion events to intracellular signaling and cytoskeletal remodeling [35,36,37]. Although this complex has been extensively studied, most investigations have focused on its role in chronic lymphocytic leukemia, a malignancy of B cells [38,39]. In contrast, the functions of CD49d in T cells and its involvement in autoimmune disorders remain insufficiently characterized, highlighting a significant research gap [40,41]. Recent studies have identified VLA-4, particularly its α-subunit CD49d, as being modulated by interferon regulatory factor 4 (IRF4) through the induction of Ikaros in B cells, suggesting that similar regulatory mechanisms may also operate in Tcells. Furthermore, interactions between Ikaros and IRF4 indirectly influence T cell motility by coordinating differentiation, as well as the expression of homing receptors and inflammatory mediators. In this context, IRF4 directly regulates genes involved in migration, while Ikaros shapes the inflammatory milieu that modulates cellular trafficking. Overexpression of IRF4 has been implicated in immune dysregulation and the pathogenesis of autoimmune diseases [42,43,44,45,46,47,48]. The IKZF1 gene encodes Ikaros (Ikaros Family Zinc Finger Protein 1), a zinc finger transcription factor essential for immune cell development, homeostasis, and function [49,50,51]. It contains six highly conserved C_2_H_2_ zinc finger domains, with ZF4 being responsible for T cell modulation [52]. There have been quite a few isoforms of Ikaros identified, resulting from alternative splicing. Two isoforms have been shown to regulate Ikaros activity, IK-1 (described as full-length Ikaros) and IK-H (the longest Ikaros isoform containing 20 extra amino acids [53]. IK-H has been identified to regulate the binding and activity of other Ikaros isoforms [54].

The cytoskeleton of T cells consists of three main filament systems: actin filaments, microtubules, and intermediate filaments. Actin filaments form a highly dynamic network that undergoes continuous remodeling during T cell activation and migration, thereby determining cell shape and motility. Microtubules are crucial for establishing cell polarity by directing the positioning of the microtubule-organizing center, whereas intermediate filaments confer mechanical stability and structural integrity, particularly under conditions of mechanical stress [50]. Actin remodeling plays a central role in T cell activation and immune synapse formation. Upon recognition of an antigen, rapid actin polymerization occurs at the contact site, resulting in the formation of a dense filamentous actin (F-actin) network at the immunological synapse. This process is tightly regulated in space and time by Rho-family GTPases, which orchestrate actin nucleation and branching dynamics [55].

Myosins represent another important group of cytoskeletal components involved in T cell motility and activation. Myosin 1G (MYO1G) is an ATP-dependent motor protein that interacts with F-actin to generate mechanical force and intracellular tension, driving cytoskeletal remodeling and cell movement. MYOO1G has been shown to increase turning and meandering motility of T cells and to prolong T cell receptor (TCR) activation upon contact with antigen-presenting cells (APCs). This prolonged activation enhances T cell differentiation and activation, thereby promoting further cytoskeletal rearrangement [51,52,53]. Myosin IIa, encoded by MYH9, is another microfilament cytoskeleton-related protein that has been described to play a key role in the maturation of the immunological synapse and is the only myosin associated with the uropod during T cell migration [54,55].

The objective of this study was to investigate the impact of altered zinc homeostasis on key molecular components required for T cell migration. To this end, HUT78 cells were cultured under three distinct conditions: zinc-adequate (ZA), zinc-supplemented (ZS), and zinc-deficient (ZD) media. Additionally, cells were stimulated with phorbol 12-myristate 13-acetate (PMA) and calcimycin (Cal) (PMA/Cal). PMA directly activates the protein kinase C, which then activates pathways and kinases, such as nuclear factor κ-light-chain-enhancer of activated B cells (NFκB). Cal is used to increase the intracellular calcium influx. In combination, PMA/Cal cause a stimulating effect on T cells [56].

We analyzed both intracellular and surface proteins, as well as cytoskeletal elements implicated in T cell motility and activation. We hypothesized that disturbances in zinc balance, particularly zinc deficiency, impair cytoskeletal reorganization and, consequently, disrupt intra- and extravascular T cell migration. For zinc supplementation experiments, we primarily focused on short-term treatment (6 h). However, as no effects were observed, we also examined 14-day supplementation, consistent with durations commonly used in in vivo studies [57,58].

Additionally, we sought to determine whether zinc deficiency affects the formation and maturation of the immunological synapse. To address this, we assessed the expression of molecules known to participate in synapse organization and stability, including MYH9, LFA-1, TLN-1, and CD49d. Through these experiments, our goal was to elucidate how zinc homeostasis influences T cell migratory behavior and immune regulation, thereby contributing to a better understanding of the potential benefits of restoring zinc balance in individuals with immune dysregulation, such as patients with T cell-mediated autoimmune diseases and elderly individuals.

## 2. Results

### 2.1. Zinc Conditions Do Not Affect the Viability of HUT78 Cells

**Figure 1:**
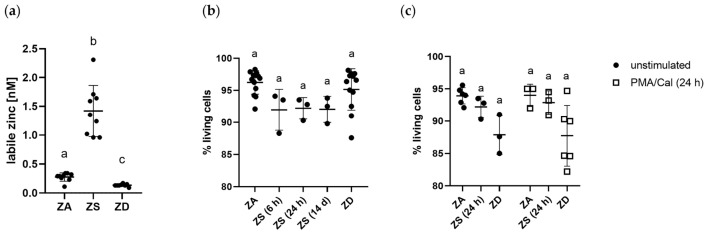
Zinc status and PMA/Cal stimulation do not influence cell viability. HUT78 cells were cultured under three conditions: zinc-adequate (ZA), zinc-deficient (ZD), and zinc-supplemented (ZS). The cells were cultured for 14 days in zinc-deficient medium. For zinc supplementation, the cells were supplemented for 6 h (ZS), 24 h (ZS (24 h)), or 14 days (ZS (14 d)). (**a**) The intracellular labile zinc concentration was measured via FluoZin-3 AM (n = 8–9). (**b**) Cell viability was assessed with PI staining for all zinc conditions used in the following experiments (n = 3–13). (**c**) HUT78 cells were activated with PMA/Cal for 24 h, and viability was assessed by PI staining after 24 h of stimulation (n = 3–6). Data is presented as mean ± SD. Statistical analysis was performed using mixed-effects analysis followed by Tukey’s test (**a**,**c**) and Kruskal–Wallis test with Dunn’s test (**b**). Significantly different results (*p* < 0.05) have no common identification letter. Groups sharing the same letter indicate no significant differences between them (**b**,**c**).

HUT78 cells were cultivated in zinc-adequate, zinc-supplemented, and zinc-deficient conditions. Intracellular labile zinc concentrations were assessed by flow cytometry using FluoZin-3 AM. Zinc supplementation (50 µM zinc sulfate) for 6 h increased the intracellular zinc concentration significantly compared to zinc-adequate conditions (*p* < 0.0001). Zinc deficiency showed a significant decrease compared to ZA (*p* = 0.0073) and ZS (*p* = 0.0003) conditions (Figure 1a). To ensure that the observed effects of zinc deficiency and supplementation represented true biological responses rather than artifacts due to reduced cell viability, cell viability was subsequently assessed using propidium iodide (PI) staining. PI staining of all zinc conditions used in the following experiments revealed that these conditions do not negatively affect cell viability (Figure 1b). Similarly, stimulation with PMA/Cal for 24 h did not alter cell viability compared to the unstimulated control (Figure 1c). Collectively, neither zinc deficiency nor the stimulation negatively impacted cell survival.

### 2.2. Zinc Deficiency Negatively Impacts the ERM Complex Expression

**Figure 2:**
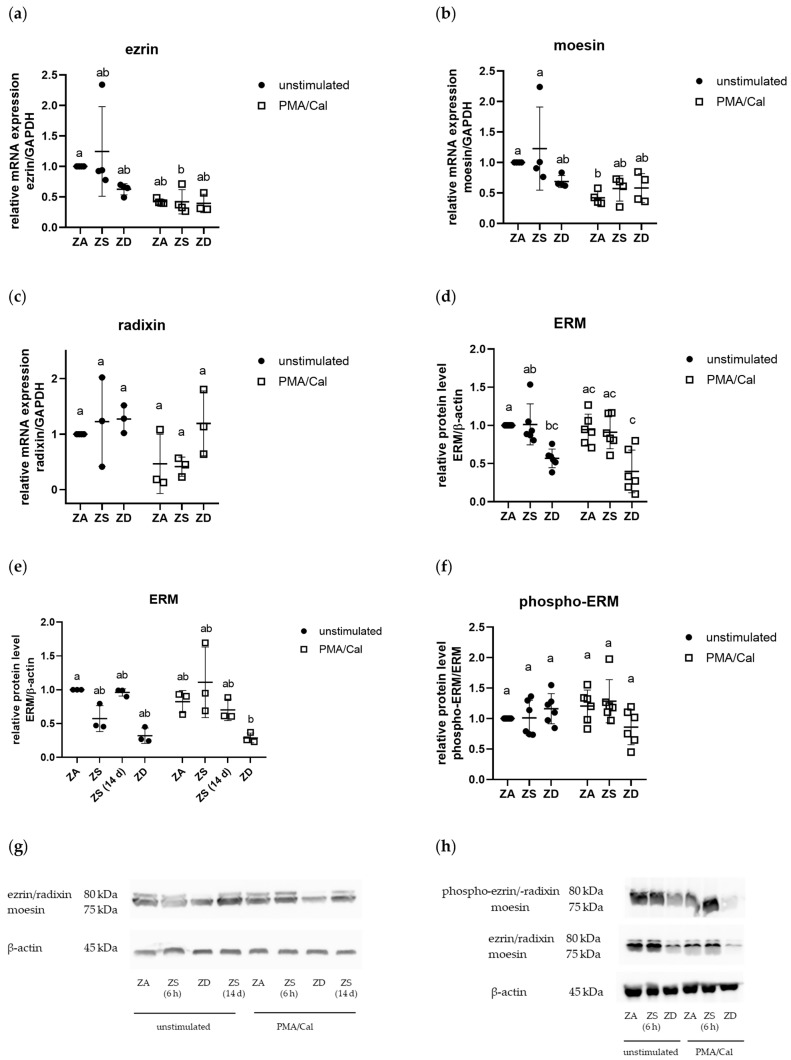
The ERM complex is negatively impacted by zinc deficiency. HUT78 cells were cultured under three conditions: zinc-adequate (ZA), zinc-deficient (ZD), and zinc-supplemented (ZS). Cells in ZA and ZD medium were maintained for 14 days. Either short-term (6 h) (**a**–**d**,**f**) or long-term (14 d) (**e**) zinc supplementation was performed. HUT78 cells were subsequently stimulated with PMA/Cal for 15 min (**a**–**d**,**f**) or 24 h (**e**). The individual components of the ERM complex were analyzed by qRT-PCR using GAPDH as the housekeeping gene, while the unphosphorylated and phosphorylated forms of the complex were examined by Western blotting with β-actin and unphosphorylated ERM as reference proteins (**d**–**f**). The mRNA expression levels of the individual ERM components are shown for ezrin (**a**) (n = 3–4), moesin (**b**) (n = 4), and radixin (**c**) (n = 3–4). (**d**) shows the protein level of unphosphorylated ERM for short-term zinc supplementation (n = 6). (**e**) HUT78 cells were supplemented with zinc for an extended period of 14 days, after which the ERM protein level was measured (n = 3). (**f**) The phosphorylation of ERM was analyzed for short-term zinc supplementation (n = 6). (**g**) An exemplary Western blot is shown for ERM and β-actin, as the data is shown in (**e**). (**h**) An exemplary Western blot for phosphor-ERM, ERM, and β-actin is shown, referring to the data presented in (**d**,**f**). Data is presented as mean ± SD. Statistical analysis was determined with Kruskal–Wallis test with Dunn’s test (**a**), Friedman test with Dunn’s test (**b**,**d**–**f**), and mixed-effects analysis with Tukey’s test (**c**). Statistical significance was defined as *p* < 0.05; groups lacking a shared identification letter indicate significant differences.

Ezrin, moesin, and radixin can exist in two conformations: a folded, inactive form maintained through F-actin interaction via the FERM domain, and an unfolded, active form that occurs when this interaction is disrupted. We examined how zinc affects the mRNA expression levels of these proteins. Upon zinc deficiency, ezrin mRNA expression was reduced. Zinc supplementation had no effect, whereas PMA/Cal stimulation and zinc supplementation decreased mRNA expression compared to zinc-adequate conditions significantly (*p* = 0.0434) (Figure 2a). A similar trend was observed for moesin in unstimulated cells. Under zinc-adequate conditions, stimulation resulted in a significant decrease compared to zinc-adequate and zinc-supplemented cells without stimulation (both *p* = 0.0375) (Figure 2b). Radixin mRNA expression remained unaffected (Figure 2c). Following the qRT-PCR analysis, Western blotting was performed to examine the effect of zinc on protein levels. Zinc deficiency had an even stronger impact at the protein level than at the mRNA level, resulting in a significantly reduced protein abundance in the unstimulated (*p* = 0.0304) and stimulated (*p* = 0.0103) cells compared to zinc-adequate conditions (Figure 2d). Because short-term zinc supplementation did not affect ERM protein expression compared to zinc-adequate conditions, we extended the treatment period. However, 14 days of zinc supplementation did not alter ERM protein levels. Consistent with earlier findings, zinc deficiency significantly reduced ERM protein expression upon prolonged PMA/Cal stimulation compared to unstimulated zinc-adequate cells (*p* = 0.0432) (Figure 2e). Phosphorylated ERM protein levels were not affected by either zinc supplementation or deficiency (Figure 2f).

### 2.3. Zinc Deficiency Decreases Focal Adhesion Kinase Expression

**Figure 3:**
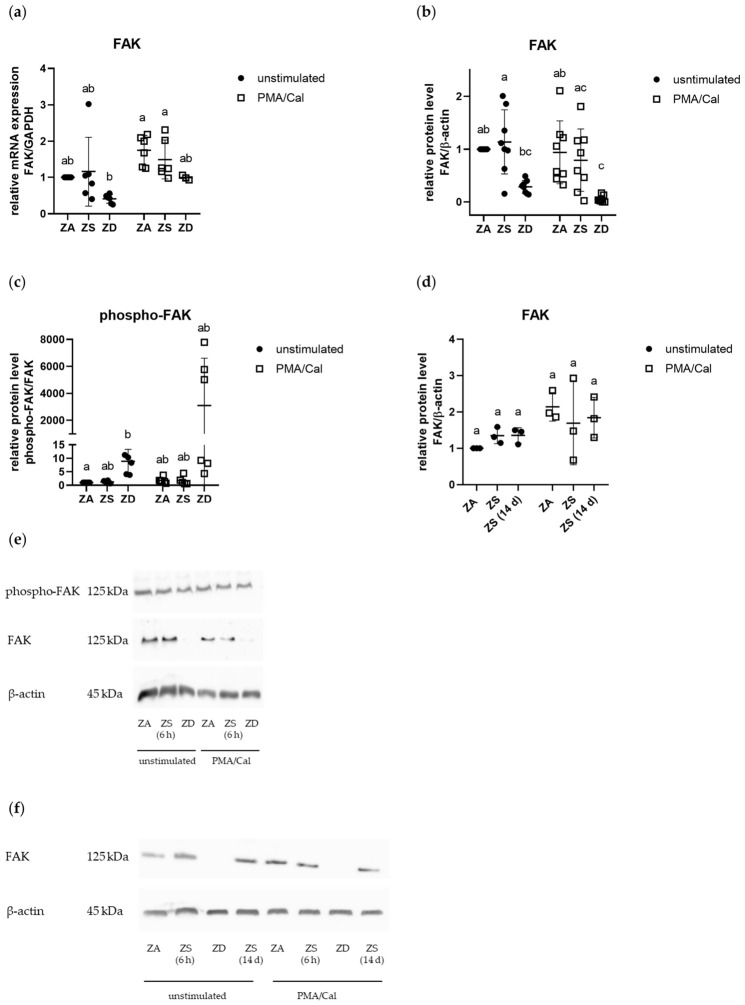
Unphosphorylated and phosphorylated FAK are impacted by zinc deficiency. HUT78 cells were cultured in zinc-adequate (ZA), zinc-deficient (ZD), or zinc-supplemented (ZS) medium. ZA and ZD conditions were maintained for 14 days, while short-term ZS treatment lasted 6 h. Additionally, cells were either not stimulated or stimulated with PMA/Cal for 15 min (**a**–**c**) or 24 h (**d**). (**a**) mRNA expression of FAK is shown (n = 3–7). (**b**) Western blot was then performed, analyzing FAK protein levels (n = 8). (**c**) Additionally, the phosphorylated form of FAK was analyzed via Western blot (n = 5–6). (**d**) The protein levels of FAK are presented for long-term zinc supplementation (14 days) (n = 3). For qRT-PCR, GAPDH was used as the housekeeping gene. For Western blotting, unphosphorylated FAK was normalized to β-actin, while phosphorylated FAK was normalized to total FAK. Representative Western blots are shown in (**e**,**f**). Data is presented as mean ± SD. Statistical differences were assessed by Kruskal–Wallis test with Dunn’s test (**a**), Friedman test with Dunn’s test (**b**), mixed-effect analysis with Tukey’s test (**c**), and one-way ANOVA with Tukey’s test (**d**). Significantly different results (*p* < 0.05) have no common identification letter.

To investigate whether zinc impacts T cell motility, we measured mRNA and protein expression levels and FAK phosphorylation in cells cultured in zinc-deficient and zinc-supplemented media. Our data showed that zinc deficiency greatly impacts FAK expression, with zinc-deficient cells expressing less FAK than zinc-adequate cells at the mRNA and protein levels. For mRNA expression, a significant difference was detected for unstimulated zinc-deficient cells compared to stimulated zinc-adequate (*p* = 0.0003) and zinc-supplemented cells (*p* = 0.0057). FAK protein levels showed significant differences in zinc-supplemented cells compared to zinc-deficient cells (*p* = 0.04933) and stimulated zinc-deficient cells (*p* = 0.00009). Additionally, stimulated zinc-deficient cells had significantly lower FAK protein expression compared to unstimulated zinc-adequate cells (*p* = 0.0077) and stimulated zinc-adequate cells (*p* = 0.0077) (Figure 3a,b). Interestingly, zinc appears to have an opposite effect on FAK phosphorylation. Zinc-deficient cells display significantly higher levels of phosphorylated protein than zinc-adequate cells (*p* = 0.0463) (Figure 3c). Cell activation through PMA/calcium stimulation appears to intensify the impact of zinc deficiency on FAK phosphorylation, resulting in elevated phospho-FAK levels when zinc-deficient cells are activated (Figure 3c). As zinc supplementation (6 h) did not show a modulating effect on FAK expression, cells were incubated for 14 days in zinc-supplemented medium. However, no significant difference for zinc-supplemented cells (14 days) compared to zinc-adequate cells could be detected (Figure 3d).

### 2.4. Zinc Deficiency Upregulates CD49d Surface Expression

**Figure 4:**
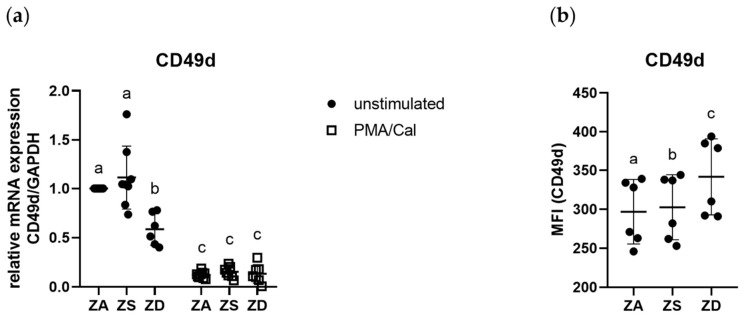
Zinc deficiency upregulates CD49d surface expression. HUT78 cells were cultured under three conditions: zinc-adequate (ZA), zinc-deficient (ZD), and zinc-supplemented (ZS). Cells were maintained in zinc-adequate and zinc-deficient media for 14 days, whereas zinc supplementation was applied for 6 h. (**a**) In addition to the zinc treatments, cells were stimulated with PMA/Cal for 15 min, and CD49d mRNA expression was quantified by qRT-PCR using GAPDH as a housekeeping gene (n = 6–9). (**b**) CD49d surface expression was determined by flow cytometry and is shown as the mean of fluorescence intensity (MFI), as all cells were CD49d positive. The experiment was conducted in ZA, ZS, and ZD conditions (n = 6). Data is presented as mean ± SD. Statistical analysis was conducted with mixed-effects analysis with Tukey’s test (**a**) and RM one-way ANOVA with Tukey’s test (**b**). Results were considered statistically significant at *p* < 0.05. Groups that differ significantly do not share a common identification letter.

We investigated the impact of altered zinc homeostasis on CD49d mRNA levels and surface expression. Zinc deficiency led to a significantly lower mRNA expression. Regardless of the zinc status, activation with PMA/Cal significantly decreased mRNA expression of CD49d (Figure 4a). Additionally, zinc-deficient cells showed a significantly lower mRNA expression of CD49 compared to zinc-adequate (*p* = 0.007) and zinc-supplemented cells (*p* = 0.033). CD49d expression on the cell surface increased significantly when cells were cultivated in a zinc-deficient medium compared to zinc-adequate (*p* = 0.001) and zinc-supplemented (*p* = 0.0036) conditions (Figure 4b).

### 2.5. Zinc Modulates the Expression of Transcription Factors Important for T Cell Migration

Zinc is known to regulate the expression of different transcription factors, such as RORyt, which is a key driver of Th17 cell differentiation [59]. Given this regulatory role, we sought to determine whether zinc availability could influence T cell migration by affecting upstream transcriptional programs involved in T cell activation and differentiation.

**Figure 5:**
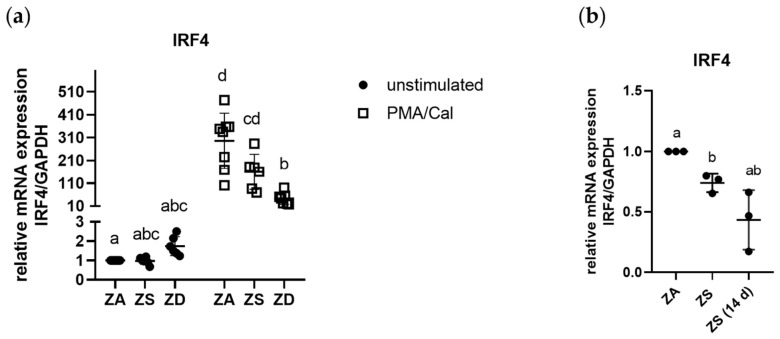
IRF4 expression is downregulated by zinc supplementation. HUT78 cells were cultured in zinc-adequate (ZA), zinc-deficient (ZD), or zinc-supplemented (ZS) medium. ZA and ZD conditions were maintained for 14 days, while short-term ZS treatment lasted 6 h (**a**) or 14 days (**b**). (**a**) Cells were either not stimulated or stimulated with PMA/Cal for 15 min. IRF4 mRNA expression was quantified by qRT-PCR (n = 6–8). (**b**) IRF4 mRNA expression of short-term (6 h) and long-term (24 h) zinc supplementation (n = 3) without PMA/Cal stimulation. GAPDH was used as a housekeeping gene. Data is presented as mean ± SD. Statistical significance was determined by mixed-effects analysis with Tukey’s test (**a**) and RM one-way ANOVA with Tukey’s test (**b**). Significantly different results (*p* < 0.05) have no common identification letter.

Therefore, we examined the expression of IRF4, a transcription factor that regulates key pathways involved in T cell migration and immunological synapse formation, under varying zinc conditions. IRF4 mRNA expression was not affected under various zinc conditions. Stimulation with PMA/Cal increased the mRNA expression of IRF4. Significant differences could be seen for all stimulated zinc conditions compared to zinc-adequate conditions without stimulation (Figure 5a). In contrast, upon stimulation, zinc deficiency induced the opposite effect observed in unstimulated cells, leading to a significant decrease in IRF4 mRNA expression compared to zinc-adequate (*p* = 0.0108) and zinc-supplemented conditions (*p* = 0.0205) (Figure 5a). Long-term zinc supplementation (14 days) resulted in a further reduction in IRF4 mRNA expression. However, a significant difference was only detectable for short-term zinc supplementation compared to the zinc-adequate condition (*p* = 0.0492) (Figure 5b).

**Figure 6:**
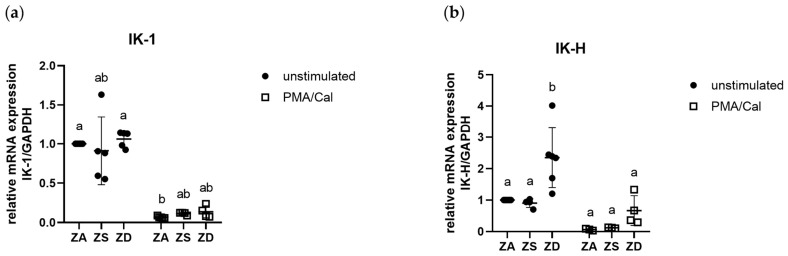
Expression of IK-H, but not IK-1, is upregulated under zinc-deficient conditions. Ikaros isoforms IK-1 and IK-H were analyzed in HUT78 cells cultured in zinc-adequate (ZA), zinc-deficient (ZD), or zinc-supplemented (ZS) medium. (**a**) ZA and ZD conditions were maintained for 14 days, while ZS treatment lasted 6 h. Following incubation, all groups were either left unstimulated or stimulated with PMA/Cal for 15 min before analysis. Relative mRNA expression was determined by qRT-PCR. GAPDH served as a housekeeping gene. (**a**) Relative mRNA expression of IK-1 is shown (n = 4–5). (**b**) IK-H expression (n = 3–6). Statistical analysis was performed by using the Kruskal–Wallis test followed by Dunn’s test (**a**) and mixed-effects analysis with Tukey’s test (**b**). Data is presented as mean ± SD. Significantly different results (*p* < 0.05) have no common identification letter.

Next, we examined the effect of zinc on another important regulator of lymphocyte differentiation, Ikaros, by quantifying the mRNA expression of its major isoforms, IK-1 and IK-H. IK-1 encodes the full-length, transcriptionally active form of Ikaros, while IK-H represents a shorter splice variant that may differ in its regulatory activity. Under zinc-adequate conditions, PMA/Cal stimulation resulted in a significant reduction in IK-1 mRNA expression compared with unstimulated zinc-adequate (*p* = 0.0051) and zinc-deficient cells (*p* = 0.0024). In contrast, stimulation of cells cultured under zinc-supplemented or zinc-deficient conditions did not significantly alter IK-1 expression (Figure 6a). IK-H expression is significantly upregulated under ZD conditions (Figure 6b).

### 2.6. Zinc Deficiency Negatively Impacts LFA-1 Subunits

Another objective of this study was to examine whether alterations in zinc homeostasis affect LFA-1. To address this, we analyzed both subunits of LFA-1, CD18 and CD11a.

**Figure 7:**
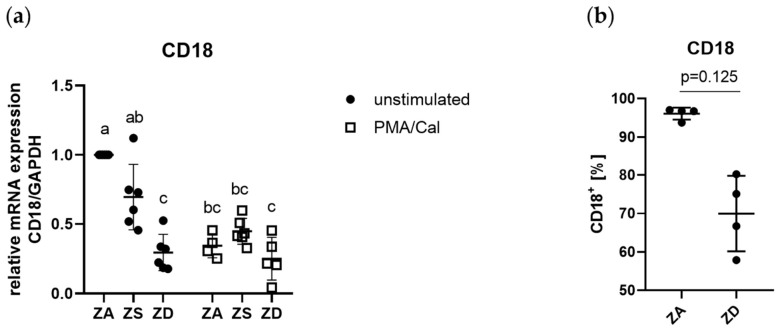
Zinc deficiency negatively impacts CD18 expression. HUT78 cells were cultured under three conditions: zinc-adequate (ZA), zinc-deficient (ZD), and zinc-supplemented (ZS). Cells were maintained in zinc-adequate and zinc-deficient media for 14 days, while zinc supplementation was applied for 6 h. (**a**) Following this incubation period, all cell groups were left either unstimulated or stimulated with PMA/Cal for 15 min prior to measurement. CD18 mRNA expression was measured via qRT-PCR. GAPDH served as a housekeeping gene (n = 4–6). (**b**) The surface expression of CD18 was analyzed via flow cytometry in ZA and ZD conditions (n = 4). Data is presented as mean ± SD. Statistical analysis was performed by using mixed-effects analysis with Tukey’s test (**a**) and by using the Wilcoxon test (**b**). Significantly different results (*p* < 0.05) have no common identification letter.

CD18 mRNA and surface expression were analyzed. Zinc deficiency showed significantly lower levels of mRNA expression compared to zinc-adequate culture conditions (*p* = 0.0003). Overall, cells stimulated independent of their zinc status showed significantly lower mRNA expression levels compared to the ZA group (Figure 7a). Furthermore, surface expression of CD18 is reduced in ZD compared to ZA (*p* = 0.125) (Figure 7b).

**Figure 8:**
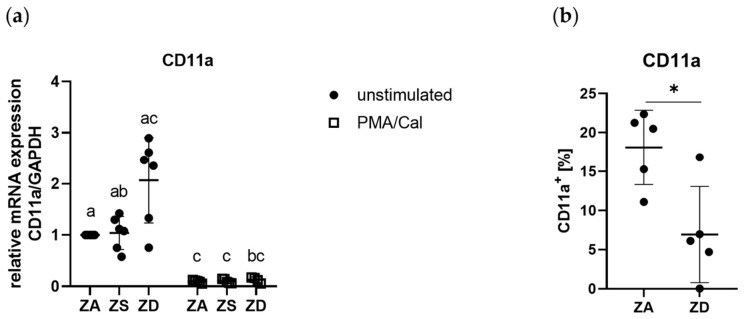
Zinc deficiency modulates CD11a expression. HUT78 cells were cultured under three conditions: zinc-adequate (ZA), zinc-deficient (ZD), and zinc-supplemented (ZS). Cells were maintained in zinc-adequate and zinc-deficient media for 14 days, while zinc supplementation was applied for 6 h. (**a**) In addition to the various zinc conditions, the cells were treated with PMA/Cal for 15 min. CD11a mRNA levels were analyzed via qRT-PCR. GAPDH served as a housekeeping gene (n = 4–6). (**b**) CD11a surface expression was determined by flow cytometry in ZA and ZD conditions (n = 5). Data is presented as mean ± SD. Statistical analysis was conducted using mixed-effects analysis with Tukey’s test (**a**) and paired two-tailed *t*-tests for (**b**) (* *p* < 0.05). Results were considered statistically significant at *p* < 0.05. Groups that differ significantly do not share a common identification letter.

To investigate whether changes in zinc homeostasis impact CD11a expression, we followed the same experimental procedure as used for CD18. Zinc deficiency led to an increased mRNA expression, while zinc supplementation had no impact. Activating the cells with PMA/Cal significantly decreased mRNA expression, regardless of zinc status, compared to unstimulated zinc-adequate conditions. However, activated cells showed no significant differences between the three stimulated groups (Figure 8a). We further investigated the surface expression of CD11a via flow cytometry. Contrary to mRNA expression levels, zinc deficiency led to a significant decrease in surface expression of CD11a (*p* = 0.0263) (Figure 8b).

### 2.7. Zinc Deficiency Seems to Impact the Expression of Several Surface Molecules

We next analyzed the expression of CD29 (β1 integrin), CD54 (ICAM-1), and CD50 (ICAM-3), additional integrins and adhesion molecules relevant to T cell migration and activation.

**Figure 9:**
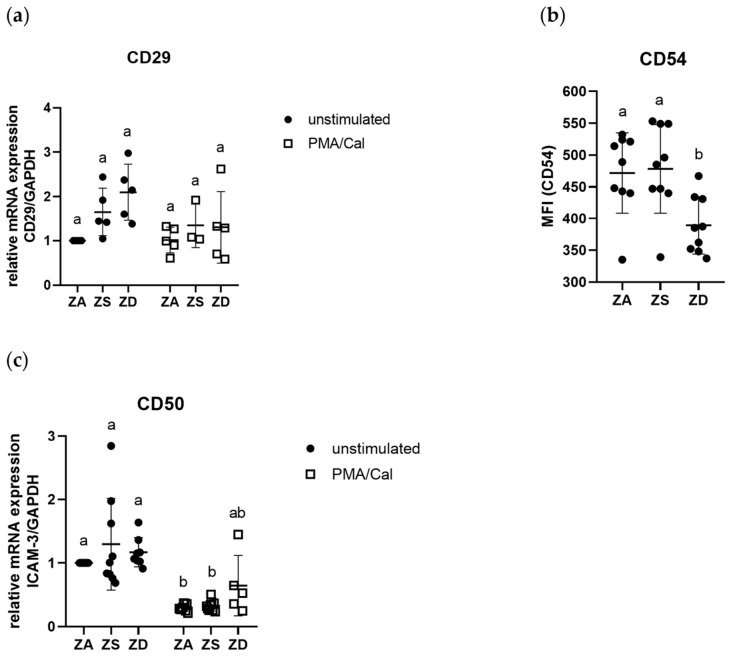
Zinc deficiency negatively affects the surface expression of CD54. HUT78 cells were cultured under three conditions: zinc-adequate (ZA), zinc-deficient (ZD), and zinc-supplemented (ZS). Cells in ZA and ZD media were maintained for 14 days, while zinc supplementation was applied for 6 h. Following zinc treatment, cells were additionally stimulated with PMA/Cal for 15 min, and mRNA expression of CD29 (n = 3–5) (**a**) and CD50 (n = 5–9) (**c**) was quantified by qRT-PCR using GAPDH as a housekeeping gene. (**b**) Surface expression of CD54 was analyzed via flow cytometry. The MFI is depicted as all cells were CD54 positive (n = 9). Data is presented as mean ± SD. Statistical analysis was conducted by using mixed-effects analysis with Tukey’s test (**a**,**c**) and RM one-way ANOVA with Tukey’s test (**b**). Results were considered statistically significant at *p* < 0.05. Groups that differ significantly do not share a common identification letter.

CD29 was neither affected by zinc status nor by stimulation with PMA/Cal (Figure 9a). Zinc deficiency led to significantly lower expression of ICAM-1 at the surface level compared to zinc-adequate (*p* = 0.0096) and zinc-supplemented (*p* = 0.0122) conditions, and supplementation had no effect (Figure 9b). For CD50 expression, the zinc status did not affect the mRNA expression. However, stimulation with PMA/Cal reduced expression (Figure 9c).

### 2.8. Talin-1 Seems to Be Unaffected by Zinc Deficiency

**Figure 10:**
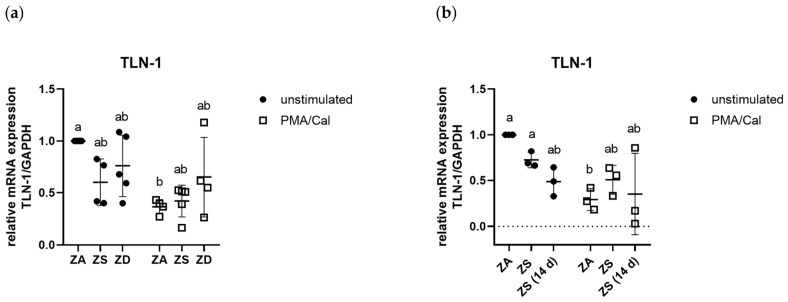
Expression of TLN-1 is impacted by longer zinc supplementation. In this experimental design, TLN-1 was investigated in HUT78 cultivated under either zinc-adequate (ZA), zinc-deficient (ZD), or zinc-supplemented (ZS) conditions. The cultivation in zinc-adequate and zinc-deficient media was carried out for 14 days; zinc supplementation was then carried out for 6 h (ZS) or for 14 days (ZS (14 d)). Additionally, cells were left either unstimulated or stimulated with PMA/Cal for 15 min prior to measurement. (**a**) TLN-1 mRNA expression is shown for ZA, ZS for 6 h, and ZD, with and without stimulation (n = 4–5). (**b**) TLN-1 mRNA expression is shown for ZA, ZS for 6 h, and ZS for 14 days, with and without stimulation (n = 3). Data is presented as mean ± SD. Statistical analysis was performed using Kruskal–Wallis test with Dunn’s test (**a**) and RM one-way ANOVA with Tukey’s test (**b**). Significantly different results (*p* < 0.05) have no common identification letter.

To further examine zinc’s impact on FAK and cytoskeletal rearrangement, we analyzed TLN-1 mRNA expression in cells cultured under different media conditions. Cells cultivated under zinc-supplemented and zinc-deficient conditions showed no significant difference in TLN-1 mRNA expression compared to the zinc-adequate control (Figure 10a). In contrast, stimulation with PMA/Cal led to a significant reduction in TLN-1 mRNA levels relative to the unstimulated zinc-adequate group (*p* = 0.0331). Since short-term zinc supplementation did not induce notable changes, cells were cultivated in zinc-supplemented medium for an extended period. Under these conditions, TLN-1 mRNA expression was significantly reduced, with the lowest levels observed after 14 days of cultivation (Figure 10b).

### 2.9. Zinc Influences Several Components of the Cytoskeleton

The T cell cytoskeleton consists of several components, including F-actin, microtubules, and intermediate filaments. The highly regulated interplay of these components is essential for the cell’s ability to reorganize the cytoskeleton, form a leading edge, and develop a uropod for migration and homing. To investigate the effects of zinc on these components, we analyzed the expression of two key proteins involved in the stability and rearrangement of the T cell cytoskeleton.

**Figure 11:**
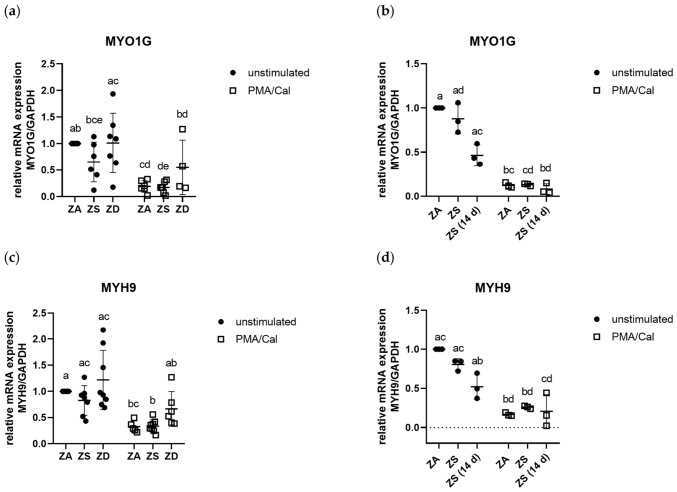
Long-term zinc supplementation negatively impacts the mRNA expression of MYO1G and MYH9. In this experimental design, MYO1G and MYH9 were investigated in HUT78 cultivated under either zinc-adequate (ZA), zinc-deficient (ZD), or zinc-supplemented (ZS) conditions. The cultivation in zinc-adequate and zinc-deficient media was carried out for 14 days; zinc supplementation was then carried out for 6 h (ZS) or for 14 days (ZS (14 d)). Additionally, cells were left either unstimulated or stimulated with PMA/Cal for 15 min prior to measurement. MYO1G (**a**) and MYH9 (**c**) mRNA expression was determined for short-term zinc supplementation ((**a**) n = 4–7; (**c**) n = 5–8). MYO1G (**b**) and MYH9 (**d**) mRNA expression was analyzed over a longer zinc supplementation period (14 days) ((**b**,**d**) n = 3). mRNA levels were determined via qRT-PCR. GAPDH served as a housekeeping gene. Data is presented as mean ± SD. Statistical analysis was performed using mixed-effects analysis (**a**,**c**) or RM one-way ANOVA (**b**,**d**) with Tukey’s test. Significantly different results (*p* < 0.05) have no common identification letter.

We investigated the mRNA expression of MYO1G and MYH9, both members of the myosin protein family, under unstimulated and stimulated zinc conditions. We found that zinc deficiency did not significantly affect the expression of either gene. However, we noticed a tendency for cells supplemented with zinc for six hours to exhibit decreased expression of both genes (Figure 11a,c). Significantly lower gene expression of MYO1G was detected for stimulated zinc-adequate (*p* < 0.0001) and stimulated zinc-supplemented (*p* < 0.0001) cells compared to unstimulated zinc-adequate cells (Figure 11a).

We then expanded the experimental design to include cells cultivated in a zinc-supplemented medium for 14 days (Figure 11b,d). These cells exhibited a further decrease in mRNA expression compared to cells supplemented with zinc for six hours. Activation of the cells with PMA/Cal decreased MYO1G and MYH9 mRNA expression in all groups (Figure 11).

**Figure 12:**
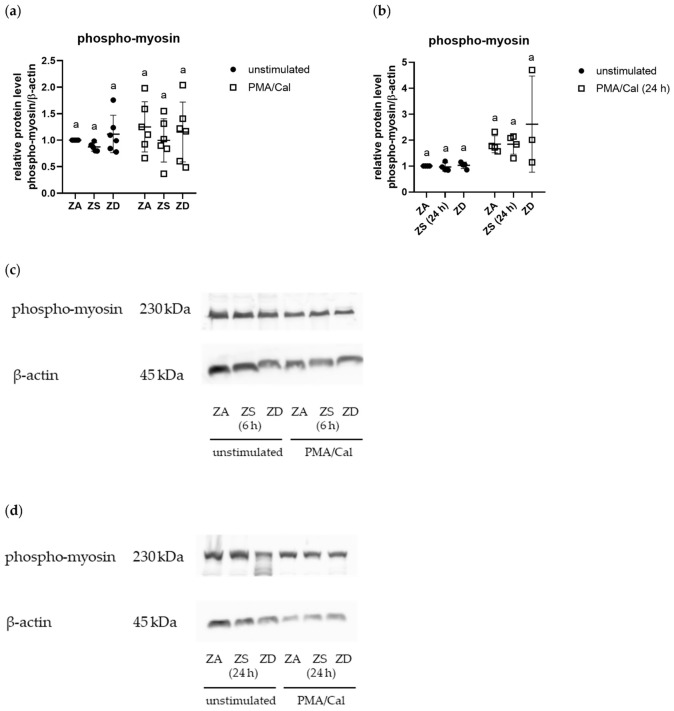
Phosphorylation of myosin is not affected by either short-term or long-term zinc supplementation. HUT78 cells were cultured under three conditions: zinc-adequate (ZA), zinc-deficient (ZD), and zinc-supplemented (ZS). Cells in ZA and ZD medium were maintained for 14 days. Either short-term (6 h) (n = 5–6) (**a**) or long-term (24 h) (n = 3–4) (**b**) zinc supplementation was performed. Cells were subsequently stimulated with PMA/Cal for 15 min (**a**) or 24 h (**b**), respectively. The relative protein level of phosphorylated myosin normalized to β-actin is shown. Corresponding Western blots are shown in (**c**,**d**). Statistical analysis was performed by using mixed-effects analysis followed by Tukey’s test (**a**,**b**). Data is presented as mean ± SD. Statistical significance was defined as *p* < 0.05; groups lacking a shared identification letter indicate significant differences.

Following the analysis of zinc effects on myosin mRNA expression, Western blotting was performed to determine whether zinc influenced phosphorylated myosin protein levels. Neither zinc deficiency, short-term zinc supplementation (6 h), nor PMA/Cal stimulation resulted in significant changes in phospho-myosin levels (Figure 12a). The experiment was extended with two additional groups treated with zinc or PMA/Cal for 24 h. Zinc supplementation for 24 h did not alter protein expression, whereas 24-h PMA/Cal stimulation resulted in a significant increase in protein levels (Figure 12b).

## 3. Discussion

Previous research has addressed the role of zinc homeostasis in the immune system. Zinc homeostasis regulates immune function, including T cell signaling, proliferation, and apoptosis [60,61]. In the adaptive immune system, zinc deficiency has been proven to impair T cell-mediated immune responses [62,63]. Autoimmune diseases such as multiple sclerosis are characterized by increased intra- and extravascular T-cell accumulation and activity [64]. To clarify how zinc shapes adaptive immunity, we investigated how altered zinc homeostasis affects T-cell motility by analyzing zinc-dependent changes in the T-cell cytoskeleton and in the expression of adhesion molecules involved in intravascular and extravascular T-cell migration. After receiving a stimulatory signal, T cells activate and rearrange their cytoskeleton to form a leading edge (chemopod) and trailing edge (uropod), whose contractility drives LFA-1 reclustering, adhesion, homing, and crawling locomotion via repetitive phosphorylation cycles [65,66]. To elucidate the role of zinc in T cell migration, we examined how altered zinc homeostasis impacts uropod components.

Prior to these analyses, intracellular zinc levels were measured using FluoZin-3 AM to confirm the intracellular zinc status after cultivation in zinc-deficient and zinc-supplemented conditions. Intracellular labile Zn^2+^, specifically measured with FluoZin-3 AM, represents the bioavailable fraction of zinc in a cell, while total zinc measurements would not capture these changes as proteins functionally quench cellular zinc [67,68]. Furthermore, PI staining verified cell viability across all conditions. As zinc is known to be a potent inhibitor of apoptosis, no further viability tests were performed [69,70].

The uropod of T cells is rich in ERM proteins, which organize membrane domains via cytoskeleton and actin interactions [71]. Previous research suggests that ezrin and moesin are required for efficient T cell adhesion and homing to lymphoid organs [72]. Our data show decreased ERM expression in zinc-deficient cells. As dephosphorylation of ERM proteins is required for T cell adhesion, zinc deficiency may impair adhesion by altering ERM phosphorylation [73]. However, the analysis of phospho-ERM/ERM in Western blot did not show significant differences. Equal phosphorylation levels indicate unchanged activation state despite reduced substrate availability, suggesting balanced phosphatase activity under zinc deficiency. Our findings further support previous research suggesting that zinc plays a major role when it comes to phosphorylation processes in T cells [74,75].

ERM proteins link LFA-1 to the actin cytoskeleton in the T cell uropod, enabling its contraction, while mechanical signals from this interaction are transduced by FAK to regulate detachment and forward movement. FAK has been suggested to be activated and phosphorylated through interactions with ezrin through its FERM domain [76,77]. Furthermore, suppressed expression of moesin limits the autophosphorylation of FAK [78]. This is supported by our findings, as mRNA expression of moesin is downregulated and protein expression of total ERM is significantly decreased in zinc deficiency. Therefore, we hypothesized that FAK would also be impacted by an altered zinc homeostasis, especially by zinc deficiency. The non-phosphorylated form of FAK was severely decreased on both mRNA and protein levels when cells were cultivated in zinc-deficient medium. However, the phosphorylated form of FAK showed a significant increase when the cells were zinc-deficient. One potential explanation is that reduced total FAK protein in zinc-deficient cells limits overall phosphorylation levels despite activation, resulting in lower phospho-FAK compared to controls. While this pattern could be interpreted as compensatory hyperactivation of FAK signaling, this conclusion remains tentative. Reduced total FAK levels may influence the apparent phosphorylation status, and in the absence of kinetic analyses or functional validation of downstream signaling, mechanistic interpretation is limited. In addition, alternative explanations, such as changes in protein stability or differences in antibody accessibility, cannot be excluded. Further studies are required to clarify the underlying mechanisms and functional consequences of these observations.

Following the effect of zinc deficiency on ERM and FAK, we investigated additional factors linked to T cell migration, particularly adhesion molecules such as CD49d, which co-localizes with FAK [30,37]. As a subunit of VLA-4 integrin, CD49d plays a key role in T cell adhesion and forms a complex with CD44 that provides mutual access to ezrin and FAK [79,80]. This complex has recently been suggested as a therapeutic target in leukemia treatment [35]. Our data showed that zinc-deficient cells expressed significantly less CD49d mRNA, further supporting the theory that zinc deficiency impairs T cell adhesion. However, surface expression of CD49d showed the opposite effect, increasing expression in zinc deficiency. While post-transcriptional regulation may contribute to these differences, no direct experimental evidence was obtained in the present study to support this mechanism. Alternative explanations, such as altered protein trafficking, turnover, or membrane localization, should also be considered.

We further investigated the effect of zinc on IRF4, a key player in the regulation of CD49d via the CD49d-IKZF1 axis [42]. Previous research suggested that increased IRF4 expression has an oncogenic capacity, including immunosuppressive effects [81,82,83]. We hypothesized that zinc deficiency leads to an increase in IRF4 expression, as it is known that IRF4 and CD49d are inversely correlated. In line with this, zinc-deficient cells expressed more IRF4 mRNA. Short-term zinc supplementation led to a significant decrease in mRNA expression, suggesting that zinc could have positive effects on keeping the oncogenic and immunosuppressive effects of IRF4 in check. Long-term zinc supplementation showed a further decrease in IRF4 mRNA expression.

Since IRF4 is known to regulate CD49d through Ikaros induction, we also investigated the possible effects of altered zinc homeostasis on Ikaros. Ikaros has also been linked to regulating FAK activation in patients with acute lymphoblastic leukemia [84]. Zinc was expected to exert similar effects on Ikaros as observed for IRF4. Neither zinc supplementation nor deficiency impacted the mRNA expression of IK-1 (full-length isoform). For IK-H, the shorter splice variant, zinc deficiency was found to increase mRNA expression, showing a similar trend to that observed for IRF4. Our research suggests that while zinc deficiency might lead to an increase in inflammation and dysfunction of T cell motility, especially adhesion, zinc supplementation might be beneficial for patients with dysregulated T cell responses, such as patients suffering from multiple sclerosis or other T cell-mediated autoimmune disorders. Although we observed zinc-dependent changes in the IRF4 and Ikaros (IK-H) isoforms, the direct mechanistic role of these changes in cytoskeletal and adhesion alterations requires further investigation in future studies.

We also investigated a possible effect of zinc on the LFA-1. LFA-1 has been described to play a major role in regulating T cell activation and migration as well as the adhesion of T cells through interacting with ICAM-1 (CD54) [85]. The LFA-1/ICAM-1 T cell adhesion is augmented by low-affinity LFA-1/ICAM-3 (CD50) interactions due to the resulting redistribution of LFA-1 [86]. While the crucial role of LFA-1 in T cell entry into lymph nodes and tissues, as well as in promoting interactions with APCs, has long been recognized, recent research highlights additional functions. These include facilitating T cell-T cell communication, supporting long-lasting T cell memory, and contributing to T cell polarization [87,88]. Recent research has linked LFA-1 to many autoimmune and inflammatory disorders, even suggesting LFA-1 as a possible therapeutic target [89,90]. Our data indicates that zinc deficiency leads to the downregulation of both CD18 and CD11a surface expression. Conversely, zinc deficiency leads to an increase in CD11a mRNA, which is possibly a compensatory mechanism for the decreased surface expression. However, this interpretation remains speculative, as no direct evidence for such a mechanism was obtained in the present study. Alternative explanations, including post-transcriptional regulation or altered protein trafficking, cannot be excluded.

In view of our results on the uropod and LFA-1, we proceeded to investigate further integrins involved in T cell adhesion, such as CD29, CD54, and CD50. CD50 has been shown to play a role in T cell adhesion by interacting with LFA-1 and enhancing VLA-4 [91,92]. Our data did not show that altered zinc homeostasis affects CD50 expression. Given the impact of zinc deficiency on CD49d expression, we decided to investigate the potential influence of zinc on VLA-4 by examining CD29, the second subunit of VLA-4. CD29 has been suggested to support the migration of memory T cells to the site of inflammation [93]. Our data demonstrated that CD29 expression remained unaffected, possibly a compensatory reaction to decreased CD49d expression to balance the expression and functionality of VLA-4. Further research is needed to identify the underlying mechanisms of apparent VLA-4 impairment in cells cultivated in zinc-deficient medium.

Given that altered zinc homeostasis affects T cell adhesion molecules, which indirectly impacts T cell receptor activation, we hypothesized that zinc might influence the formation and maturation of the immunological synapse. Our current data on LFA-1 strongly suggest that immunological synapse formation is impaired by zinc deficiency because LFA-1 plays a crucial role in its formation [94,95]. The formation of the immunological synapse has also been suggested to be modulated by FAK through TCR-FAK interactions [20]. We have shown that zinc deprivation also lowers FAK expression and thus could further hamper the formation of an immunological synapse.

Since zinc appears to affect the expression of several elements involved in the formation and maturation of the immunological synapse, we decided to investigate the possible effects of zinc on other components linked to the immunological synapse, such as class I and II myosins and TLN-1. We demonstrated that zinc supplementation, particularly long-term, led to decreased expression of TLN-1. Considering that TLN-1 overexpression has been linked to increased aggressiveness and metastasis in several types of cancer, we suggest that zinc supplementation may positively impact the negative effects of TLN-1 overexpression [96,97,98,99].

MYO1G regulated membrane tension by interacting with F-actin. Lower expression of MYO1G has been linked to faster migration and shorter interactions between T cells and APCs [100,101]. MYO1G overexpression has been linked to a poor prognosis and increased aggressiveness of acute lymphoblastic leukemia [102,103]. We hypothesized that altered zinc homeostasis might have an impact on the expression of MYO1G. However, we demonstrated that altered zinc homeostasis did not affect MYO1G gene expression significantly. Only with long-term zinc supplementation was a trend toward decreased mRNA expression observed.

The heavy chain of non-muscle myosin II (MYH9) maintains T cell uropods and motility via LFA-1 adhesion, while also driving immunological synapse maturation, microcluster fusion, and persistence [24,104,105,106]. Overexpression of MYH9 has been suggested to play a part in inadequate T cell responses in cancer patients [107,108]. Seeing the effect of zinc on other components related to the uropod and the immunological synapse, we hypothesized that zinc might also have an effect on MYH9. Our research demonstrated that long-term zinc supplementation decreases MYH9 expression, like MYO1G. These results imply that zinc supplementation could balance myosin overexpression, thereby stabilizing the immunological synapse and promoting appropriate T cell responses.

We conclude that zinc plays an important role in T cell migration at multiple levels, especially in T cell adhesion. Our study showed that zinc deficiency impairs the formation of the uropod and LFA-1 function, which could hinder the crawling of T lymphocytes and their entrance into lymphoid organs, leading to compromised adaptive immunity. Additionally, zinc deficiency could undermine the formation and maturation of the immunological synapse.

However, there are some limitations that should be noted. All experiments were performed in vitro using a human T cell line under controlled zinc-deficient and zinc-supplemented conditions, which may not fully capture the complex in vivo microenvironment, including interactions with other immune cells or fluctuating systemic zinc levels. Furthermore, the use of the HUT78 T-cell line in combination with pharmacological stimulation using PMA/calcimycin limits the physiological interpretability of the results, as this approach can adversely affect key processes such as integrin signaling, cytoskeletal reorganization, and the formation of immunological synapses. While our data demonstrate clear effects on mRNA and protein expression and surface markers, functional consequences (e.g., functional migration assays, synapse stability, or chemotaxis) require validation. Zinc repletion was not assessed, as stable in vitro repletion remains technically challenging due to medium-dependent free Zn^2+^ bioavailability. Furthermore, while ex vivo experiments with primary T cells would provide additional validation, zinc deficiency is known to directly impair their function and differentiation capacity, as demonstrated in primary human T cells [109]. Our HUT-78 findings thus recapitulate these clinically relevant phenotypes while eliminating donor variability inherent to primary cell studies. Additionally, observed discrepancies between mRNA and surface expression (e.g., CD49d) suggest post-transcriptional regulation that warrants further mechanistic investigation.

Despite these limitations, our results suggest that restoring zinc homeostasis could support important functions of the adaptive immune system, such as cell migration to the site of infection and communication with other immune cells. However, as these findings are based on in vitro experiments, their relevance to in vivo immune responses remains uncertain. Further in vivo studies and experiments using primary human cells are needed to validate these observations under physiological conditions and to clarify their potential implications for autoimmune diseases. Clinical studies will further be required to determine whether zinc supplementation is beneficial in patients with T cell-mediated autoimmune disorders or in populations with low serum zinc levels, such as the elderly, in whom enhanced T cell proliferation has previously been reported following zinc supplementation [110,111,112,113,114]. Our in vitro findings supplement previous research suggesting beneficial effects of zinc supplementation on mRNA and protein expression involved in T cell migration. Furthermore, we propose that restoring normal zinc homeostasis could improve the T cell response after vaccination; however, further investigation is required. Notably, zinc deficiency negatively impacts CD49d and CD11a, which enhance the detection of antigen-specific T cells following human vaccination [115].

## 4. Materials and Methods

### 4.1. Cell Culture and Stimulation

HUT78 cells were cultivated in RPMI-1640 medium (both from Sigma Aldrich, Steinheim, Germany), which contained 10% fetal calf serum (FCS) (Bio&Sell GmbH, Nürnberg, Germany), 2 mM L-glutamine, 100 U/mL potassium penicillin, and 100 µg/mL streptomycin sulfate (all from Sigma-Aldrich). Cells were cultivated in zinc-adequate, zinc-deficient, or zinc-supplemented RPMI-1640 medium. To achieve zinc deficiency, the cells were cultivated for 14 days in zinc-deficient medium. Zinc-deficient medium was made using Chelex 100 beads (Bio-Rad Laboratories Inc., Hercules, CA, USA) following the manufacturer’s instructions and contained 0.3 µM zinc or less. Calcium and magnesium were reconstituted as described before [116]. The zinc-adequate medium contained 7 µM zinc, and for zinc supplementation, the medium was supplemented with 50 µM zinc sulfate (Sigma Aldrich). The zinc concentrations were determined by atomic absorption spectroscopy (AAS) as described previously [59,60]. We performed experiments with short-term zinc supplementation with 50 µM zinc sulfate for 6 h and long-term supplementation for 24 h or 14 days.

HUT78 cells were then stimulated with 10 ng/mL PMA (Sigma Aldrich) and 1 µM calcimycin (Sigma Aldrich) for 15 min or 24 h.

### 4.2. Western Blot

After zinc treatment and additional stimulation, Western blot samples were prepared. Supernatants were discarded, and cell pellets were resuspended in sample buffer (65 mM Tris-HCl (Sigma-Aldrich), 25% glycerol (Fisher Scientific GmbH, Schwerte, Germany), 2% SDS, 0.01% bromophenol blue, 1% β-mercaptoethanol, 1 mM sodium orthovanadate (all from Sigma-Aldrich)). Samples were sonicated for 30 s and heated up for 5 min at 95 °C for protein denaturation. Samples were then separated using sodium-dodecyl-sulfate polyacrylamide gel electrophoresis (8.5% polyacrylamide gel) next to a blue-colored protein standard on each gel (New England Biolabs, Frankfurt, Germany). Samples were then blotted to a nitrocellulose membrane (Amersham, Piscataway, NJ, USA) and then blocked for at least one hour in tris-buffered saline (TBS: 20 mM Tris-HCl, 136 mM NaCl) (Applichem, Darmstadt, Germany), which was supplemented with 0.1% Tween 20 (Sigma-Aldrich) with 5% non-fat dry milk (Saliter, Obergünzburg, Germany). After blocking, the nitrocellulose membranes were incubated in the primary antibody overnight at 4 °C. Antibodies were bought from Cell Signaling Technologies (Danvers, MA, USA) and used as recommended by the manufacturer: β-actin (#4967), phospho-myosin (#3675), ERM (#3142), phospho-ERM (#3726), FAK (#3285), phospho-FAK (#8556). Incubation with secondary anti-rabbit antibody (Cell Signaling Technologies) was completed for at least two hours at room temperature after the membrane had been washed with TBS-T. The secondary antibody was diluted 1:1000 in TBS-T supplemented with 5% non-fat dry milk. Subsequently, luminol enhancer solution and peroxide (Westar Antares, Cyanagen, Bologna, Italy) were added to the membranes for five minutes, and the membranes were stored in a dark environment until detection. Brightfield and chemiluminescence images were made with LAS 3000 (Fujifilm Lifesciences, Düsseldorf, Germany) and the blots were quantified using ImageJ 1.54g (National Institutes of Health, Bethesda, MD, USA). Molecular weight markers on the blots were imaged under brightfield conditions. Chemiluminescent detection of target proteins was subsequently performed on the same membrane without repositioning.

### 4.3. RNA Isolation and cDNA Reverse Transcription

The RNA isolation from HUT78 cells was completed using the EXTRACTME total RNA KIT (Blirt, Gdansk, Poland) according to the manufacturer’s instructions. The total amount of RNA was measured using a NanoDrop ND 1000 spectrophotometer (Thermo Fisher Scientific, Maltham, MA, USA). Then, 1 µg RNA was reverse-transcribed with the qScript cDNA synthesis kit (Quantabio, Beverly, MA, USA) following the manufacturer’s protocol. The obtained cDNA was then used for SYBR-green-based qPCR (Quant Studio 3, Thermo Fisher). Primer sequences and temperatures were used as described in Table 1. GAPDH served as a housekeeping gene. Data were analyzed by $2^{-∆∆Ct}$ method.

### 4.4. Flow Cytometry

The zinc-adequate and zinc-deficient cells were stained with propidium iodide (10 µg/mL) to analyze cell viability.

HUT78 cells were stained with the antibodies CD11a, CD18, CD54, and CD49d (Cell Signaling Technologies) as described previously [33]. The samples were measured with flow cytometry (FACSCalibur, BD Sciences, Heidelberg, Germany) while the data were analyzed with Cellquest Software 3.0 (BD Sciences). An exemplary gating strategy is provided in Supplementary Figure S1.

### 4.5. Measurement of Intracellular Labile Zinc Concentration

HUT78 were incubated with 1 µM FluoZin-3 AM (Invitrogen, Thermo Fisher Scientific, Eugene, OR, USA), a membrane-permeable and zinc-responsive fluorescent dye, prepared in phosphate-buffered saline (PBS; Sigma-Aldrich). Following a 30-min incubation at 37 °C, the cells were washed twice with PBS. The fluorescence intensity of the labeled cells was then measured directly to obtain the baseline signal (F).

To establish the lower and upper fluorescence limits required for quantifying free intracellular Zn^2+^, the cells were exposed either to 50 µM TPEN to achieve minimal fluorescence (Fmin) or to 100 µM zinc sulfate with 5 µM pyrithione to reach maximal fluorescence (Fmax). These treatments were carried out for 15 min at 37 °C, after which the fluorescence was measured by flow cytometry (FACSCalibur, BD Sciences) as described previously [117].

The intracellular concentration of freely available Zn^2+^ was calculated based on the known dissociation constant (K_d_) of FluoZin-3 AM (8.9 nM) using Equation (1):

Intracellular labile zinc [nM] = K_d_ × ((F − Fmin))/((Fmax − F)) (1)

An exemplary gating strategy is provided in Supplementary Figure S1.

### 4.6. Statistics

For each experiment, at least three independent experiments (n ≥ 3) were performed, with outliers identified using the ROUT method (Q = 1%). Normality was assessed using the Shapiro–Wilk test (n ≤ 8) or Anderson–Darling test (n > 8). Statistical significance was determined based on data distribution: RM one-way ANOVA or mixed-effects models (for repeated measures data with missing values) with Tukey’s post hoc test (parametric), Friedman test with Dunn’s post hoc test, or Kruskal–Wallis test with Dunn’s post hoc test (non-parametric). Greenhouse–Geisser correction was applied for every experiment to account for sphericity violations in repeated measures designs. A *t*-test was used for a two-group comparison.

Data is presented as mean ± SD. When the mean of each column was compared with the mean of every other column, letters were used to clearly show significant differences. Significantly different results (* *p* < 0.05) have no common identification letter. For *t*-tests, significances are indicated as * *p* < 0.05. Statistical tests were performed using GraphPad Prism Software (San Diego, CA, USA) version 8.0.0 for Windows. Additionally, the descriptive statistic for each plot is presented in the Supplementary Materials.

## Figures and Tables

**Table 1:** Primer sequences. The primer sequences for each target gene, along with their corresponding annealing temperatures, are listed below.

Target Gene	Forward Primer (5′→3′)	Reverse Primer (5′→3′)	Annealing Temperature
CD18	AGACTGGTAGCAAAGCCCCC	CACTCCTGAGAGAGGACGCAC	60 °C
CD29	CCGCGCGGAAAAGATAA	CACAATTTGGCCCTGCTTGTA	60 °C
CD49d	GATGCTGTTCTGTGCCTG	CCCACTAGGAGCCATCGGTT	62 °C
CD50	GCTGCGAGTCCTGTATGGTC	GGCTGGAGCCTTCCTTCAAA	60 °C
ezrin	CTCGGCGGACGCAAGG	ACTCGGACATTGATTGGTTTCG	60 °C
FAK	TGTGGGTAAACCAGATCCTGC	CTGAAGCTTGACACCCTCGT	60 °C
GAPDH	GAA GGT GAA GGT CGG AGT C	GAAGAT GGTGATGGGATTTC	60 °C
IK-1	CCTGGGCGGGACGCT	TGGTCCTCAGGTTATGTGGATTT	60 °C
IK-H	GATCCCTCCCAGGTGGCT	CATGGTCCTCAGGTTATCAACCAAC	60 °C
moesin	ACGCTAGTGAGGGACCCAAT	ACGCACACTGATCGTTTTGG	60 °C
MYH9	AGGTGTCCCATCTCTTGGGT	ATGGCAAAGTCAGCCTGCTC	60 °C
MYO1G	CTACCTACTGGAGAAGTCTCGG	AGCAGGGTTTCTCTCCAAGTG	60 °C
radixin	TGAGTTTGAAGCAATGTGGGG	GGACTCTTGTCTCCACTCTAGC	60 °C
TLN-1	CTCCTGAGTGACTCGCTTCC	CAGTTCTGTGGCTGCCTGAT	60 °C

## Data Availability

The original contributions presented in this study are included in the article/Supplementary Material. Further inquiries can be directed to the corresponding author.

## Supplementary Materials

The following supporting information can be downloaded at https://www.mdpi.com/article/10.3390/ijms27125249/s1.

## Abbreviations

The following abbreviations are used in this manuscript:

AAS	Linear dichroism
AAS	Atomic absorption spectroscopy
APC	Antigen-presenting cell
Cal	Calcimycin
ERM	Ezrin, radixin, and moesin
F-actin	Filamentous actin
FAK	Focal adhesion kinase
FCS	Fetal calf serum
ICAM-1	Intracellular adhesion molecule-1
ICAM-3	Intracellular adhesion molecule-3
IRF4	Interferon regulatory factor 4
LFA-1	Lymphocyte function-associated antigen 1
MFI	Mean of fluorescence intensity
Phospho	phosphorylated
PI	Propidium iodide
PMA	phorbol 12-myristate 13-acetate
qRT-PCR	Quantitative real-time polymerase chain reaction
TCR	T cell receptor
TLN-1	Talin-1
VCAM-1	Vascular cell adhesion molecule-1
VLA-4	Very late antigen-4
ZA	Zinc-adequate
ZD	Zinc-deficient
ZS	Zinc-supplemented
