# Supplementary material for: The Impact of Zinc on T Cell Motility and the Immunological Synapse
Source: Int J Mol Sci. 2026 Jun 10;27(12):5249. doi: 10.3390/ijms27125249 (PMC13299819; doi:10.3390/ijms27125249)
Supplement: Supplementary file 1 [file ijms-27-05249-s001.zip › ijms-4249966-supplementary.pdf]

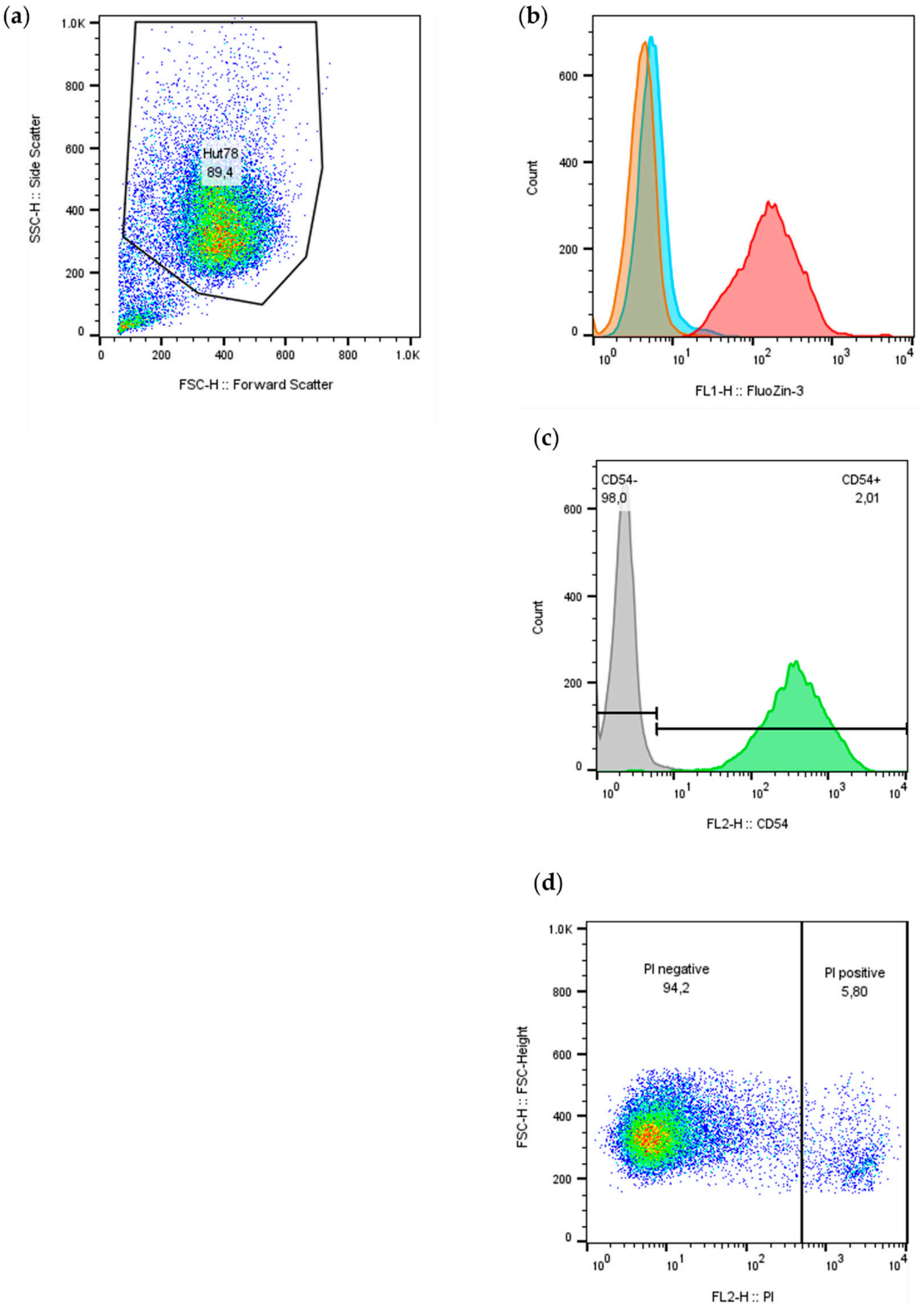

Supplementary Figure S1. Exemplary gating strategies of HUT78. (a) This panel shows an exemplary gating of the cell population before any further analysis. (b) FluoZin-3 AM staining was performed, and fluorescence peaks are shown from a representative experiment (orange: Fmin, blue: F, red: Fmax). (c) CD54 staining (green) was performed with the respective isotype control (grey). Cells were gated as positive using a 2% false positive threshold, and the mean fluorescence intensity (MFI) was used for further analysis. (d) PI staining was performed, and the frequency of PI-negative cells (% parent) was used for viability analysis.

Supplementary Table S1. Descriptive data from Figure 1 a) with the number of replicates (n), median, interquartile, mean, and standard deviation.

	ZA	ZS (6 h)	ZD
	unstimulated		
Number of independent replicates (n)	9	9	8
Minimum	0.1100	0.9600	0.09000
25% Percentile	0.2450	0.9900	0.1300
Median	0.2900	1.350	0.1300
75% Percentile	0.3300	1.675	0.1475
Maximum	0.3400	2.310	0.1700
Range	0.2300	1.350	0.08000
Mean	0.2756	1.420	0.1338
Std. Deviation	0.07161	0.4436	0.02264

Supplementary Table S2. Descriptive data from Figure 1 b) with the number of replicates (n), median, interquartile range, mean, and standard deviation.

	ZA	ZS (6 h)	ZS (24 h)	ZS (14 d)	ZD
	unstimulated				
Number of independent replicates (n)	13	3	3	3	12
Minimum	92.10	88.30	90.30	89.80	87.60
25% Percentile	94.75	88.30	90.30	89.80	93.06
Median	96.89	93.50	92.80	92.50	96.49
75% Percentile	97.54	94.10	93.50	93.80	97.52
Maximum	98.25	94.10	93.50	93.80	98.13
Range	6.150	5.800	3.200	4.000	10.53
Mean	96.25	91.97	92.20	92.03	95.14
Std. Deviation	1.829	3.190	1.682	2.040	3.229

Supplementary Table S3. Descriptive data from Figure 1 c) with the number of replicates (n), median, interquartile range, mean, and standard deviation.

	ZA	ZS (24 h)	ZD	ZA	ZS (24 h)	ZD
	Unstimulated			PMA/Cal (24 h)		
Number of independent replicates (n)	6	3	3	3	3	6
Minimum	92.10	90.30	85.00	92.00	90.90	82.20
25% Percentile	92.71	90.30	85.00	92.00	90.90	84.00
Median	94.10	92.80	87.60	95.00	93.20	87.32
75% Percentile	94.99	93.50	91.00	95.00	94.50	91.40
Maximum	95.57	93.50	91.00	95.00	94.50	94.69
Range	3.470	3.200	6.000	3.000	3.600	12.49
Mean	93.93	92.20	87.87	94.00	92.87	87.74
Std. Deviation	1.257	1.682	3.009	1.732	1.823	4.689

Supplementary Table S4. Descriptive data from Figure 2 a) with the number of replicates (n), median, interquartile range, mean, and standard deviation.

	ZA	ZS	ZD	ZA	ZS	ZD
	Unstimulated			PMA/Cal		
Number of independent replicates (n)	4	4	4	4	4	3
Minimum	1.000	0.7785	0.4930	0.3991	0.2681	0.2991
25% Percentile	1.000	0.8152	0.5314	0.4002	0.2833	0.2991
Median	1.000	0.9316	0.6548	0.4076	0.3512	0.3140
75% Percentile	1.000	1.990	0.6899	0.4663	0.6276	0.5686
Maximum	1.000	2.341	0.6989	0.4846	0.7122	0.5686
Range	0.000	1.563	0.2059	0.08548	0.4441	0.2694
Mean	1.000	1.246	0.6254	0.4247	0.4207	0.3939
Std. Deviation	0.000	0.7339	0.09091	0.04026	0.1991	0.1515

Supplementary Table S5. Descriptive data from Figure 2 b) with the number of replicates (n), median, interquartile range, mean, and standard deviation.

	ZA	ZS	ZD	ZA	ZS	ZD
	Unstimulated			PMA/Cal		
Number of independent replicates (n)	4	4	4	4	4	4
Minimum	1.000	0.7637	0.6188	0.3292	0.2720	0.3641
25% Percentile	1.000	0.7989	0.6256	0.3348	0.3562	0.3731
Median	1.000	0.9555	0.6484	0.3894	0.6491	0.5589
75% Percentile	1.000	1.930	0.7892	0.5399	0.7180	0.8125
Maximum	1.000	2.238	0.8352	0.5775	0.7276	0.8441
Range	0.000	1.475	0.2165	0.2483	0.4556	0.4800
Mean	1.000	1.228	0.6877	0.4214	0.5744	0.5815
Std. Deviation	0.000	0.6808	0.09936	0.1122	0.2076	0.2363

Supplementary Table S6. Descriptive data from Figure 2 c) with the number of replicates (n), median, interquartile range, mean, and standard deviation.

	ZA	ZS	ZD	ZA	ZS	ZD
	Unstimulated			PMA/Cal		

Number of independent replicates (n)	4	3	3	3	3	3
Minimum	1.000	0.4124	1.020	0.1294	0.2273	0.6406
25% Percentile	1.000	0.4124	1.020	0.1294	0.2273	0.6406
Median	1.000	1.237	1.277	0.1841	0.4497	1.137
75% Percentile	1.000	2.020	1.515	1.083	0.5668	1.803
Maximum	1.000	2.020	1.515	1.083	0.5668	1.803
Range	0.000	1.608	0.4946	0.9533	0.3395	1.162
Mean	1.000	1.223	1.271	0.4654	0.4146	1.193
Std. Deviation	0.000	0.8041	0.2474	0.5353	0.1725	0.5833

Supplementary Table S7. Descriptive data from Figure 2 d) with the number of replicates (n), median, interquartile range, mean, and standard deviation.

	ZA	ZS	ZD	ZA	ZS	ZD
	Unstimulated			PMA/Cal		
Number of independent replicates (n)	6	6	6	6	6	6
Minimum	1.000	0.8051	0.3863	0.7094	0.6074	0.1015
25% Percentile	1.000	0.8551	0.4842	0.7578	0.7562	0.1704
Median	1.000	0.9092	0.5717	0.9317	0.8644	0.3023
75% Percentile	1.000	1.170	0.6440	1.111	1.158	0.7146
Maximum	1.000	1.534	0.7597	1.265	1.163	0.7991
Range	0.000	0.7293	0.3734	0.5559	0.5553	0.6976
Mean	1.000	1.013	0.5686	0.9452	0.9102	0.3975
Std. Deviation	0.000	0.2679	0.1223	0.2007	0.2161	0.2807

Supplementary Table S8. Descriptive data from Figure 2 e) with the number of replicates (n), median, interquartile range, mean, and standard deviation.

	ZA	ZS	ZS (14 d)	ZD	ZA	ZS	ZS (14 d)	ZD
	Unstimulated				PMA/Cal (24 h)			

Number of independent replicates (n)	3	3	3	3	3	3	3	3
Minimum	1.000	0.4559	0.8985	0.2436	0.6362	0.6889	0.6078	0.2329
25% Percentile	1.000	0.4559	0.8985	0.2436	0.6362	0.6889	0.6078	0.2329
Median	1.000	0.4722	0.9903	0.2686	0.9049	0.9492	0.6141	0.2615
75% Percentile	1.000	0.7957	0.9930	0.4520	0.9330	1.695	0.8865	0.3702
Maximum	1.000	0.7957	0.9930	0.4520	0.9330	1.695	0.8865	0.3702
Range	0.000	0.3399	0.09453	0.2083	0.2968	1.006	0.2786	0.1372
Mean	1.000	0.5746	0.9606	0.3214	0.8247	1.111	0.7028	0.2882
Std. Deviation	0.000	0.1917	0.05381	0.1138	0.1639	0.5221	0.1591	0.07241

Supplementary Table S9. Descriptive data from Figure 2 f) with the number of replicates (n), median, interquartile range, mean, and standard deviation.

	ZA	ZS	ZD	ZA	ZS	ZD
	Unstimulated			PMA/Cal		
Number of independent replicates (n)	6	6	6	6	6	6
Minimum	1.000	0.7346	0.8438	0.8305	0.9684	0.4481
25% Percentile	1.000	0.7410	0.9396	0.9435	1.072	0.5981
Median	1.000	0.9641	1.163	1.262	1.193	0.8844
75% Percentile	1.000	1.313	1.346	1.393	1.444	1.126
Maximum	1.000	1.364	1.548	1.555	1.977	1.194
Range	0.000	0.6296	0.7047	0.7242	1.008	0.7456
Mean	1.000	1.011	1.162	1.205	1.284	0.8603
Std. Deviation	0.000	0.2895	0.2486	0.2623	0.3545	0.2904

Supplementary Table S10. Descriptive data from Figure 3 a) with the number of replicates (n), median, interquartile range, mean, and standard deviation.

	ZA	ZS	ZD	ZA	ZS	ZD
	Unstimulated			PMA/Cal		

Number of independent replicates (n)	7	6	6	6	6	3
Minimum	1.000	0.4060	0.2545	1.249	0.9788	0.9321
25% Percentile	1.000	0.5272	0.2783	1.280	1.114	0.9321
Median	1.000	0.9404	0.4378	1.841	1.250	0.9845
75% Percentile	1.000	1.572	0.5103	2.118	2.097	1.059
Maximum	1.000	3.020	0.5624	2.189	2.310	1.059
Range	0.000	2.614	0.3079	0.9399	1.332	0.1272
Mean	1.000	1.161	0.4119	1.751	1.496	0.9919
Std. Deviation	0.000	0.9492	0.1195	0.4105	0.5378	0.06394

Supplementary Table S11. Descriptive data from Figure 3 b) with the number of replicates (n), median, interquartile range, mean, and standard deviation.

	ZA	ZS	ZD	ZA	ZS	ZD
	Unstimulated			PMA/Cal		
Number of independent replicates (n)	8	8	8	8	8	8
Minimum	1.000	0.1543	0.1407	0.3224	0.02305	0.000
25% Percentile	1.000	0.7123	0.1637	0.4596	0.2518	0.004535
Median	1.000	1.059	0.2915	0.8138	0.7616	0.03345
75% Percentile	1.000	1.731	0.3817	1.262	1.172	0.1069
Maximum	1.000	2.006	0.4861	2.105	1.806	0.1676
Range	0.000	1.851	0.3454	1.782	1.783	0.1676
Mean	1.000	1.136	0.2854	0.9394	0.7916	0.05279
Std. Deviation	0.000	0.6086	0.1216	0.5976	0.5911	0.06172

Supplementary Table S12. Descriptive data from Figure 3 c) with the number of replicates (n), median, interquartile range, mean, and standard deviation.

	ZA	ZS	ZD	ZA	ZS	ZD
	Unstimulated			PMA/Cal		

Number of independent replicates (n)	6	6	6	6	5	6
Minimum	1.000	0.7100	3.838	0.7361	0.5985	4.361
25% Percentile	1.000	0.7506	4.054	1.225	0.6077	7.203
Median	1.000	1.311	9.332	1.656	1.051	2518
75% Percentile	1.000	1.665	12.29	2.306	3.174	6275
Maximum	1.000	1.833	15.38	3.777	4.435	7792
Range	0.000	1.123	11.54	3.041	3.836	7788
Mean	1.000	1.256	8.877	1.838	1.723	3102
Std. Deviation	0.000	0.4666	4.433	1.024	1.607	3509

Supplementary Table S13. Descriptive data from Figure 3 d) with the number of replicates (n), median, interquartile range, mean, and standard deviation.

	ZA	ZS	ZS (14 d)	ZA	ZS	ZS (14 d)
	Unstimulated			PMA/Cal (24 h)		
Number of independent replicates (n)	3	3	3	3	3	3
Minimum	1.000	1.149	1.108	1.863	0.6737	1.300
25% Percentile	1.000	1.149	1.108	1.863	0.6737	1.300
Median	1.000	1.311	1.457	1.971	1.475	1.816
75% Percentile	1.000	1.590	1.497	2.589	2.927	2.412
Maximum	1.000	1.590	1.497	2.589	2.927	2.412
Range	0.000	0.4402	0.3896	0.7257	2.253	1.112
Mean	1.000	1.350	1.354	2.141	1.692	1.842
Std. Deviation	0.000	0.2227	0.2143	0.3916	1.142	0.5564

Supplementary Table S14. Descriptive data from Figure 4 a) with the number of replicates (n), median, interquartile range, mean, and standard deviation.

	ZA	ZS	ZD	ZA	ZS	ZD
	Unstimulated			PMA/Cal		

Number of independent replicates (n)	9	8	6	9	8	7
Minimum	1.000	0.7357	0.4010	0.07702	0.06515	0.005782
25% Percentile	1.000	0.8804	0.4254	0.09372	0.1102	0.06820
Median	1.000	1.042	0.5663	0.1274	0.1584	0.1072
75% Percentile	1.000	1.305	0.7677	0.1408	0.1963	0.1798
Maximum	1.000	1.760	0.7791	0.1865	0.2364	0.2951
Range	0.000	1.025	0.3781	0.1095	0.1713	0.2894
Mean	1.000	1.113	0.5850	0.1215	0.1530	0.1325
Std. Deviation	0.000	0.3224	0.1632	0.03334	0.05578	0.09330

Supplementary Table S15. Descriptive data from Figure 4 b) with the number of replicates (n), median, interquartile range, mean, and standard deviation.

	ZA	ZS	ZD
	Unstimulated		
Number of independent replicates (n)	6	6	6
Minimum	246.0	253.0	291.0
25% Percentile	258.8	259.8	291.8
Median	299.5	309.5	344.5
75% Percentile	335.3	339.5	387.3
Maximum	339.0	344.0	394.0
Range	93.00	91.00	103.0
Mean	296.8	302.7	341.8
Std. Deviation	41.30	41.67	49.09

Supplementary Table S16. Descriptive data from Figure 5 a) with the number of replicates (n), median, interquartile range, mean, and standard deviation.

	ZA	ZS	ZD	ZA	ZS	ZD
	Unstimulated			PMA/Cal		
Number of independent replicates (n)	8	6	6	8	6	7
Minimum	1.000	0.6836	1.236	100.8	69.74	17.87
25% Percentile	1.000	0.8501	1.340	183.4	82.39	20.21
Median	1.000	0.9907	1.613	341.8	169.9	43.79
75% Percentile	1.000	1.139	2.235	357.2	207.1	55.24
Maximum	1.000	1.192	2.496	473.6	283.4	90.44
Range	0.000	0.5084	1.260	372.9	213.7	72.57
Mean	1.000	0.9807	1.747	295.9	160.2	42.97
Std. Deviation	0.000	0.1788	0.4862	121.0	76.94	25.94

Supplementary Table S17. Descriptive data from Figure 5 b) with the number of replicates (n), median, interquartile range, mean, and standard deviation.

	ZA	ZS	ZD
	Unstimulated		

Number of independent replicates (n)	3	3	3
Minimum	1.000	0.6511	0.1735
25% Percentile	1.000	0.6511	0.1735
Median	1.000	0.7657	0.4662
75% Percentile	1.000	0.7962	0.6595
Maximum	1.000	0.7962	0.6595
Range	0.000	0.1451	0.4861
Mean	1.000	0.7377	0.4331
Std. Deviation	0.000	0.07652	0.2447

Supplementary Table S18. Descriptive data from Figure 6 a) with the number of replicates (n), median, interquartile range, mean, and standard deviation.

	ZA	ZS	ZD	ZA	ZS	ZD
	Unstimulated			PMA/Cal		
Number of independent replicates (n)	5	5	5	5	4	4
Minimum	1.000	0.5532	0.9234	0.05980	0.08978	0.07379
25% Percentile	1.000	0.5734	0.9525	0.05985	0.09746	0.07590
Median	1.000	0.8831	1.129	0.06616	0.1226	0.1177
75% Percentile	1.000	1.268	1.137	0.07853	0.1247	0.2165
Maximum	1.000	1.631	1.140	0.08976	0.1247	0.2376
Range	0.000	1.077	0.2168	0.02996	0.03495	0.1638
Mean	1.000	0.9131	1.062	0.06858	0.1149	0.1367
Std. Deviation	0.000	0.4322	0.1018	0.01234	0.01687	0.07612

Supplementary Table S19. Descriptive data from Figure 6 b) with the number of replicates (n), median, interquartile range, mean, and standard deviation.

	ZA	ZS	ZD	ZA	ZS	ZD
	Unstimulated			PMA/Cal		
Number of independent replicates (n)	6	4	6	3	3	4
Minimum	1.000	0.7032	1.201	0.03303	0.1029	0.2924
25% Percentile	1.000	0.7613	1.573	0.03303	0.1029	0.3099
Median	1.000	0.9377	2.365	0.06310	0.1225	0.5152
75% Percentile	1.000	1.005	2.838	0.09118	0.1319	1.166
Maximum	1.000	1.026	4.017	0.09118	0.1319	1.332
Range	0.000	0.3231	2.816	0.05815	0.02895	1.039
Mean	1.000	0.9012	2.348	0.06244	0.1191	0.6636
Std. Deviation	0.000	0.1385	0.9524	0.02908	0.01477	0.4743

Supplementary Table S20. Descriptive data from Figure 7 a) with the number of replicates (n), median, interquartile range, mean, and standard deviation.

	ZA	ZS	ZD	ZA	ZS	ZD

	Unstimulated			PMA/Cal		
Number of independent replicates (n)	6	6	6	4	6	5
Minimum	1.000	0.4560	0.1779	0.2518	0.3272	0.04294
25% Percentile	1.000	0.5025	0.1840	0.2653	0.3868	0.1241
Median	1.000	0.6643	0.2720	0.3351	0.4239	0.2158
75% Percentile	1.000	0.8396	0.3834	0.4314	0.5300	0.3951
Maximum	1.000	1.120	0.5242	0.4538	0.5967	0.4535
Range	0.000	0.6643	0.3463	0.2020	0.2695	0.4106
Mean	1.000	0.6948	0.2948	0.3439	0.4477	0.2509
Std. Deviation	0.000	0.2373	0.1308	0.08645	0.09308	0.1541

Supplementary Table S21. Descriptive data from Figure 7 b) with the number of replicates (n), median, interquartile range, mean, and standard deviation.

	ZA	ZD
	Unstimulated	
Number of independent replicates (n)	4	4
Minimum	93.74	57.88
25% Percentile	94.49	60.09
Median	96.76	70.91
75% Percentile	96.97	78.94
Maximum	97.03	80.22
Range	3.290	22.34
Mean	96.07	69.98
Std. Deviation	1.560	9.797

Supplementary Table S22. Descriptive data from Figure 8 a) with the number of replicates (n), median, interquartile range, mean, and standard deviation.

	ZA	ZS	ZD	ZA	ZS	ZD
	Unstimulated			PMA/Cal		
Number of independent replicates (n)	6	6	6	5	5	4
Minimum	1.000	0.5774	0.7561	0.05569	0.06331	0.05987
25% Percentile	1.000	0.7071	1.189	0.07170	0.06667	0.07650
Median	1.000	1.099	2.408	0.1054	0.08835	0.1453
75% Percentile	1.000	1.329	2.682	0.1226	0.1461	0.1730
Maximum	1.000	1.420	2.891	0.1292	0.1470	0.1760
Range	0.000	0.8429	2.135	0.07351	0.08373	0.1161
Mean	1.000	1.041	2.068	0.09882	0.1028	0.1316
Std. Deviation	0.000	0.3215	0.8335	0.02850	0.04062	0.05231

Supplementary Table S23. Descriptive data from Figure 8 b) with the number of replicates (n), median, interquartile range, mean, and standard deviation.

	ZA	ZD
	Unstimulated	
Number of independent replicates (n)	5	5
Minimum	11.10	0.04000
25% Percentile	13.19	2.365
Median	20.48	6.110
75% Percentile	21.81	11.90
Maximum	22.36	16.82
Range	11.26	16.78
Mean	18.09	6.926
Std. Deviation	4.761	6.144

Supplementary Table S24. Descriptive data from Figure 9 a) with the number of replicates (n), median, interquartile range, mean, and standard deviation.

	ZA	ZS	ZD	ZA	ZS	ZD
	Unstimulated			PMA/Cal		
Number of independent replicates (n)	5	5	5	5	3	5
Minimum	1.000	1.044	1.378	0.6074	1.035	0.5844
25% Percentile	1.000	1.229	1.492	0.7525	1.035	0.6428
Median	1.000	1.441	2.149	0.9920	1.079	1.284
75% Percentile	1.000	2.181	2.676	1.293	1.921	1.973
Maximum	1.000	2.441	2.977	1.321	1.921	2.620
Range	0.000	1.397	1.599	0.7133	0.8858	2.036
Mean	1.000	1.652	2.097	1.017	1.345	1.303
Std. Deviation	0.000	0.5399	0.6347	0.2900	0.4992	0.8084

Supplementary Table S25. Descriptive data from Figure 9 b) with the number of replicates (n), median, interquartile range, mean, and standard deviation.

	ZA	ZS	ZD
	Unstimulated		
Number of independent replicates (n)	9	9	9
Minimum	335.0	339.0	337.0
25% Percentile	441.5	443.5	350.0
Median	489.0	485.0	385.0
75% Percentile	522.5	549.0	432.5
Maximum	532.0	553.0	467.0
Range	197.0	214.0	130.0
Mean	471.8	478.3	389.2
Std. Deviation	63.21	69.61	45.24

Supplementary Table S26. Descriptive data from Figure 9 c) with the number of replicates (n), median, interquartile range, mean, and standard deviation.

	ZA	ZS	ZD	ZA	ZS	ZD
	Unstimulated			PMA/Cal		
Number of independent replicates (n)	9	9	8	9	9	5
Minimum	1.000	0.6862	0.9110	0.2112	0.2349	0.2471
25% Percentile	1.000	0.7934	1.026	0.2585	0.2560	0.3011
Median	1.000	1.007	1.108	0.2845	0.3188	0.5270
75% Percentile	1.000	1.798	1.312	0.3625	0.3764	1.048
Maximum	1.000	2.841	1.635	0.3700	0.5025	1.449
Range	0.000	2.155	0.7244	0.1587	0.2676	1.202
Mean	1.000	1.295	1.169	0.2976	0.3272	0.6449
Std. Deviation	0.000	0.7215	0.2301	0.05608	0.08403	0.4751

Supplementary Table S27. Descriptive data from Figure 10 a) with the number of replicates (n), median, interquartile range, mean, and standard deviation.

	ZA	ZS	ZD	ZA	ZS	ZD
	Unstimulated			PMA/Cal		
Number of independent replicates (n)	5	4	5	4	5	4
Minimum	1.000	0.4006	0.3998	0.2709	0.1648	0.2629
25% Percentile	1.000	0.4051	0.4958	0.2948	0.2780	0.3345
Median	1.000	0.5906	0.6772	0.3810	0.5086	0.5818
75% Percentile	1.000	0.8079	1.064	0.4220	0.5188	1.038
Maximum	1.000	0.8230	1.086	0.4307	0.5243	1.179
Range	0.000	0.4224	0.6858	0.1598	0.3595	0.9157
Mean	1.000	0.6012	0.7593	0.3659	0.4204	0.6513
Std. Deviation	0.000	0.2228	0.2960	0.06859	0.1528	0.3833

Supplementary Table S28. Descriptive data from Figure 10 b) with the number of replicates (n), median, interquartile range, mean, and standard deviation.

	ZA	ZS	ZS (14 d)	ZA	ZS	ZS (14 d)
	Unstimulated			PMA/Cal		
Number of independent replicates (n)	3	3	3	3	3	3
Minimum	1.000	0.6621	0.3280	0.1833	0.3324	0.02985
25% Percentile	1.000	0.6621	0.3280	0.1833	0.3324	0.02985
Median	1.000	0.6869	0.4891	0.2744	0.5540	0.1708
75% Percentile	1.000	0.8197	0.6426	0.4192	0.6348	0.8568
Maximum	1.000	0.8197	0.6426	0.4192	0.6348	0.8568
Range	0.000	0.1576	0.3146	0.2359	0.3024	0.8269
Mean	1.000	0.7229	0.4866	0.2923	0.5071	0.3525
Std. Deviation	0.000	0.08473	0.1573	0.1189	0.1566	0.4424

Supplementary Table S29. Descriptive data from Figure 11 a) with the number of replicates (n), median, interquartile range, mean, and standard deviation.

	ZA	ZS	ZD	ZA	ZS	ZD
	Unstimulated			PMA/Cal		
Number of independent replicates (n)	7	6	7	6	7	4
Minimum	1.000	0.1197	0.1769	0.01963	0.01838	0.1645
25% Percentile	1.000	0.3365	0.6366	0.1174	0.07167	0.1714
Median	1.000	0.6379	1.091	0.1779	0.1711	0.3812
75% Percentile	1.000	1.015	1.344	0.3038	0.2783	1.096
Maximum	1.000	1.130	1.934	0.3251	0.3144	1.271
Range	0.000	1.010	1.757	0.3055	0.2960	1.107
Mean	1.000	0.6518	1.012	0.1912	0.1702	0.5496
Std. Deviation	0.000	0.3761	0.5596	0.1109	0.1043	0.5156

Supplementary Table S30. Descriptive data from Figure 11 b) with the number of replicates (n), median, interquartile range, mean, and standard deviation.

	ZA	ZS	ZS (14 d)	ZA	ZS	ZS (14 d)
	Unstimulated			PMA/Cal		
Number of independent replicates (n)	3	3	3	3	3	3
Minimum	1.000	0.7267	0.3632	0.1002	0.1161	0.04018
25% Percentile	1.000	0.7267	0.3632	0.1002	0.1161	0.04018
Median	1.000	0.8479	0.4331	0.1111	0.1372	0.05155
75% Percentile	1.000	1.059	0.5970	0.1558	0.1401	0.1492
Maximum	1.000	1.059	0.5970	0.1558	0.1401	0.1492
Range	0.000	0.3324	0.2337	0.05560	0.02398	0.1090
Mean	1.000	0.8779	0.4644	0.1224	0.1311	0.08030
Std. Deviation	0.000	0.1682	0.1200	0.02948	0.01310	0.05991

Supplementary Table S31. Descriptive data from Figure 11 c) with the number of replicates (n), median, interquartile range, mean, and standard deviation.

	ZA	ZS	ZD	ZA	ZS	ZD
	Unstimulated			PMA/Cal		
Number of independent replicates (n)	8	7	8	5	8	6
Minimum	1.000	0.4333	0.6913	0.2199	0.1660	0.3860
25% Percentile	1.000	0.5218	0.7760	0.2399	0.2598	0.4010
Median	1.000	0.8825	0.9557	0.2925	0.3272	0.5710
75% Percentile	1.000	0.9624	1.806	0.4323	0.4086	0.9070
Maximum	1.000	1.271	2.175	0.4960	0.5595	1.270
Range	0.000	0.8374	1.483	0.2762	0.3936	0.8841
Mean	1.000	0.8278	1.220	0.3274	0.3391	0.6650
Std. Deviation	0.000	0.2821	0.5655	0.1089	0.1190	0.3308

Supplementary Table S32. Descriptive data from Figure 11 d) with the number of replicates (n), median, interquartile range, mean, and standard deviation.

	ZA	ZS	ZS (14 d)	ZA	ZS	ZS (14 d)
	Unstimulated			PMA/Cal		
Number of independent replicates (n)	3	3	3	3	3	3
Minimum	1.000	0.7197	0.3728	0.1524	0.2404	0.02361
25% Percentile	1.000	0.7197	0.3728	0.1524	0.2404	0.02361
Median	1.000	0.8447	0.4945	0.1579	0.2619	0.1543
75% Percentile	1.000	0.8508	0.6944	0.1912	0.2770	0.4430
Maximum	1.000	0.8508	0.6944	0.1912	0.2770	0.4430
Range	0.000	0.1312	0.3216	0.03879	0.03659	0.4194
Mean	1.000	0.8050	0.5206	0.1672	0.2597	0.2070
Std. Deviation	0.000	0.07402	0.1624	0.02100	0.01839	0.2146

Supplementary Table S33. Descriptive data from Figure 12 a) with the number of replicates (n), median, interquartile range, mean, and standard deviation.

	ZA	ZS	ZD	ZA	ZS	ZD
	Unstimulated			PMA/Cal		
Number of independent replicates (n)	6	5	6	6	6	6
Minimum	1.000	0.7954	0.7757	0.6595	0.3664	0.4858
25% Percentile	1.000	0.7969	0.8248	0.8550	0.7183	0.5741
Median	1.000	0.8641	1.044	1.180	0.9570	1.196
75% Percentile	1.000	0.9449	1.368	1.686	1.372	1.565
Maximum	1.000	0.9850	1.756	1.981	1.548	2.038
Range	0.000	0.1896	0.9807	1.322	1.182	1.552
Mean	1.000	0.8696	1.116	1.252	0.9962	1.154
Std. Deviation	0.000	0.07931	0.3557	0.4747	0.4095	0.5656

Supplementary Table S34. Descriptive data from Figure 12 b) with the number of replicates (n), median, interquartile range, mean, and standard deviation.

	ZA	ZS (24 h)	ZD	ZA	ZS (24 h)	ZD
	Unstimulated			PMA/Cal (24 h)		
Number of independent replicates (n)	4	4	4	4	4	3
Minimum	1.000	0.8422	0.8556	1.564	1.299	1.142
25% Percentile	1.000	0.8465	0.9038	1.602	1.432	1.142
Median	1.000	0.9119	1.049	1.742	1.956	2.011
75% Percentile	1.000	1.130	1.128	2.183	2.127	4.701
Maximum	1.000	1.186	1.154	2.321	2.142	4.701
Range	0.000	0.3435	0.2982	0.7571	0.8433	3.559
Mean	1.000	0.9629	1.027	1.842	1.838	2.618
Std. Deviation	0.000	0.1580	0.1244	0.3308	0.3841	1.855
